# The Division of Amyloid Fibrils: Systematic Comparison of Fibril Fragmentation Stability by Linking Theory with Experiments

**DOI:** 10.1016/j.isci.2020.101512

**Published:** 2020-08-29

**Authors:** David M. Beal, Magali Tournus, Ricardo Marchante, Tracey J. Purton, David P. Smith, Mick F. Tuite, Marie Doumic, Wei-Feng Xue

**Affiliations:** 1 Kent Fungal Group, School of Biosciences, University of Kent, CT2 7NJ Canterbury, UK; 2 Centrale Marseille, I2M, UMR 7373, CNRS, Aix-Marseille Univ., Marseille 13453, France; 3 Biomolecular Research Centre, Sheffield Hallam University, Sheffield, UK; 4 INRIA Rocquencourt, équipe-projet BANG, Domaine de Voluceau, BP 105, 78153 Rocquencourt, France; 5 Wolfgang Pauli Institute, University of Vienna, Vienna, Austria

**Keywords:** Molecular Modelling, Cell Biology

## Abstract

The division of amyloid protein fibrils is required for the propagation of the amyloid state and is an important contributor to their stability, pathogenicity, and normal function. Here, we combine kinetic nanoscale imaging experiments with analysis of a mathematical model to resolve and compare the division stability of amyloid fibrils. Our theoretical results show that the division of any type of filament results in self-similar length distributions distinct to each fibril type and the conditions applied. By applying these theoretical results to profile the dynamical stability toward breakage for four different amyloid types, we reveal particular differences in the division properties of disease-related amyloid formed from α-synuclein when compared with non-disease associated model amyloid, the former showing lowered intrinsic stability toward breakage and increased likelihood of shedding smaller particles. Our results enable the comparison of protein filaments' intrinsic dynamic stabilities, which are key to unraveling their toxic and infectious potentials.

## Highlights

• Theory on the division of amyloid fibrils developed using a continuous PDE framework
• The theory allowed direct analysis of fibril breakage properties with AFM image data
• The new insights enabled comparison of fibrils' intrinsic stability to breakage
• α-Synuclein fibrils showed low stability to division compared with other model amyloid

## Graphical Abstract

**Figure:**
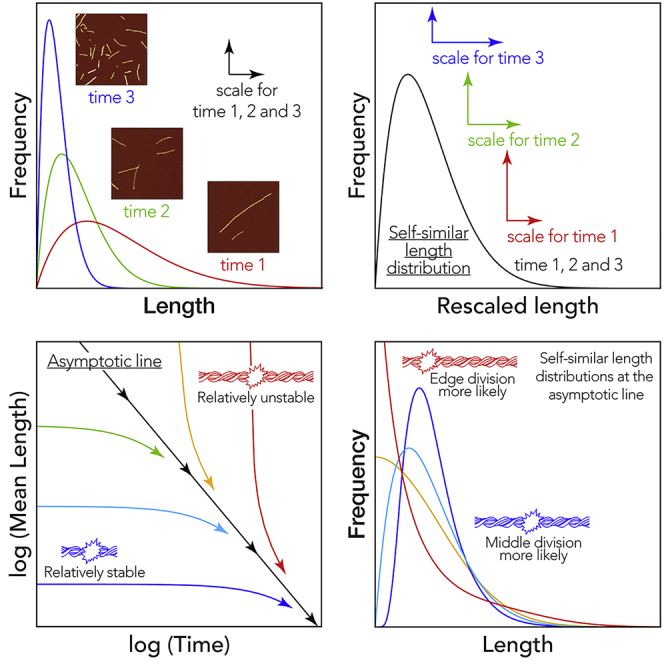

## Introduction

**Figure 1:**
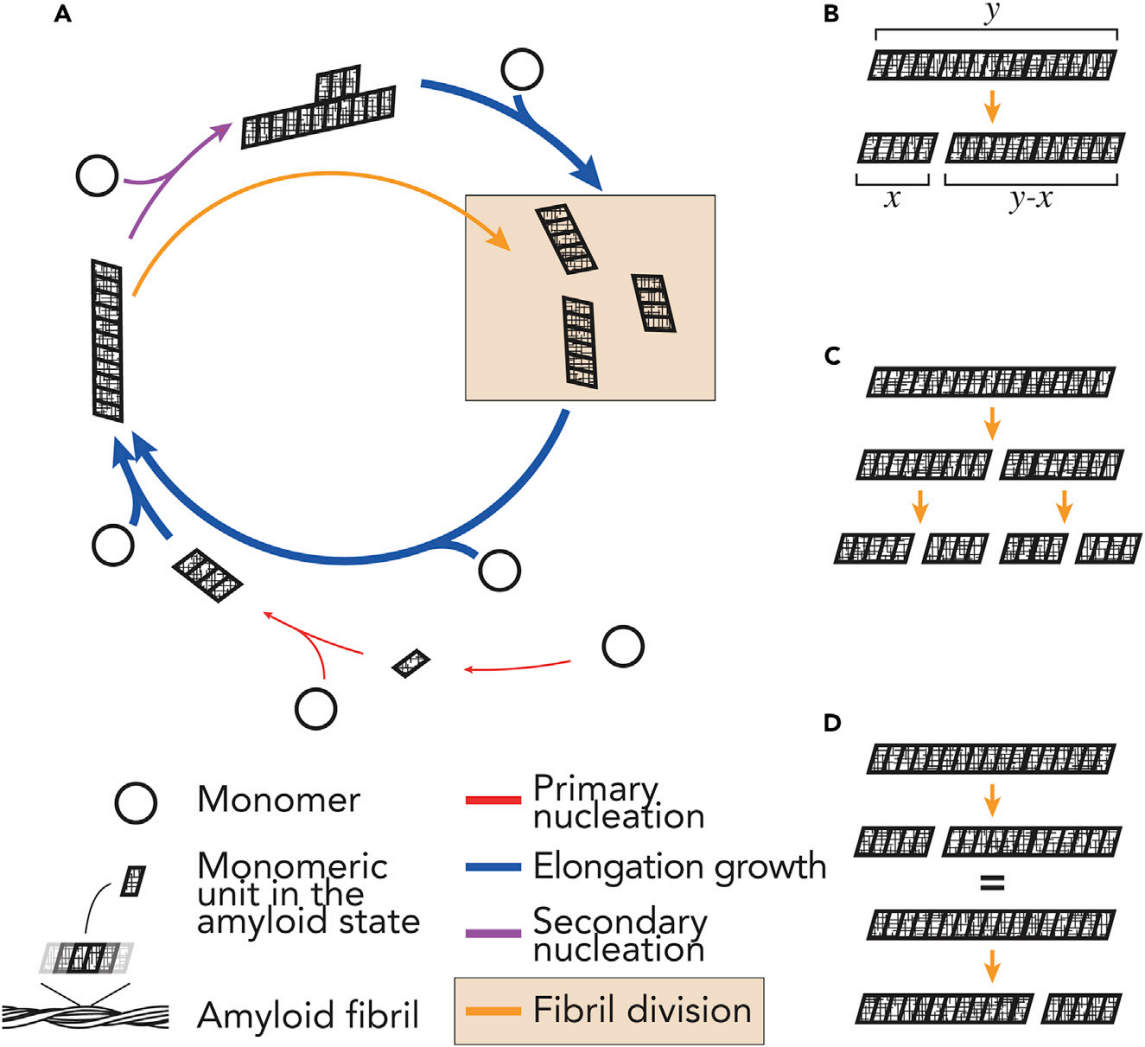
Schematic Illustration of Fibril Division in the Amyloid Life Cycle (A) The life cycle of amyloid assembly where soluble monomeric proteins (circles) are converted into the amyloid state with a cross-β conformation (the parallelograms). The colored arrows represent the four main processes in amyloid assembly. Red arrows represent primary nucleation, which may occur as homogeneous nucleation in solution and heterogeneous nucleation at interfaces. Primary nucleation may also occur subsequent to liquid-liquid phase separation or phase transitions (Khan et al., 2018). Purple arrows represent secondary nucleation, which may occur as heterogeneous nucleation at surfaces presented by preformed aggregates. Blue arrows represent growth by elongation at fibril ends. Yellow arrows and box represent fibril division (e.g., fibril fragmentation or breakage). The arrows may represent consecutive reversible steps, and the thickness of the arrows symbolizes the relative rates involved in the processes. (B) A simple model of fibril division, where a given parent fibril particle of length y divides to give rise to two daughter fibril particles of size x and y-x. The model does not otherwise identify the lineage of the individual fibrils. (C) The division model assumes that each parent fibril particle divides into exactly two daughter particles at each microscopic reaction step. (D) The division model assumes that the division rate for each microscopic step is identical as long as the resulting two particles have the same size.

Amyloid fibrils, proteinaceous polymers with a cross-β core structure, represent an important class of bio-nanomaterials (Bleem and Daggett, 2017; Knowles and Buehler, 2011). They are also important biological structures associated with devastating human diseases such as Alzheimer disease, Parkinson disease, Creutzfeldt-Jakob disease, systemic amyloidosis, and type 2 diabetes (Knowles et al., 2014), as well as have vital biological functions such as adhesion and biofilm formation, epigenetic switches, and hormone storage (e.g., Berson et al., 2003; Bleem and Daggett, 2017; Chapman et al., 2002; Knowles and Buehler, 2011; Larsen et al., 2007; Li et al., 2014; Romero et al., 2010; Tuite and Serio, 2010). Division of amyloid fibrils, which can manifest *in vitro* in amyloid nanomaterials or *in vivo* in disease-associated or functional amyloid aggregates, is mediated by mechanical agitation, thermal stress, chemical perturbation, or chaperone catalysis. Fibril division is a crucial step in the life cycle of amyloid (Figure 1A) (Xue, 2015) and enables the propagation of the amyloid protein conformation and biological information encoded therein. Despite knowledge of its importance, it is not understood why amyloid division processes give rise to varied biological impacts ranging from normal propagation of functional amyloid assemblies to large inert structures or the creation of molecular species involved in disease, e.g., small cytotoxic amyloid species and infective prions, which are transmissible amyloid particles. In this respect, the resistance of amyloid to division is also a critical aspect to protein misfolding associated with disease progression and biological roles of functional amyloid assemblies (e.g., Tanaka et al., 2006). In terms of disease association, there is much debate as to how amyloid aggregates are associated with cellular toxicity, with evidence of both prefibrillar oligomers and fibrillar species (Breydo and Uversky, 2015; Tipping et al., 2015) giving rise to disease-related phenotypes. Although it is hypothesized that all proteins can undergo conversion into an amyloid state (Dobson, 1999), why most proteins do not form amyloid under physiological conditions or produce amyloid particles that are non-toxic, non-transmissible, or non-disease associated is not clear. In this debate, it has been suggested that fibrils are not merely the end product of amyloid aggregation, but rather elicit profound biological responses through fibril fragmentation and oligomer shedding (Tipping et al., 2015), due to lack of fibril stability.

Amyloid fibrils have remarkable physical properties, such as their tensile strength comparable to that of steel and elasticity similar to spider silk (Knowles et al., 2007). As proteinaceous polymers, they also offer the potential for modification by rational design, which makes them an ideal target for the development of biologically compatible nanomaterials (Bleem and Daggett, 2017; Hu et al., 2018; Li et al., 2014; Mankar et al., 2011). This interest in amyloid as a bio-nanomaterial has led to a search for proteins and peptides that can undergo conversion into a stable amyloid conformation while lacking the properties that associate them with toxicity, infectivity, and disease. Although the precise properties that associate some amyloid to disease or biological function are not resolved, the potential for different morphologies (sometimes referred to as “strains”) to elicit different results (Meinhardt et al., 2009; Sachse et al., 2010; Tanaka et al., 2006) could be attributed to the stability of amyloid fibrils toward division or their mechanical properties (Marchante et al., 2017; Xue et al., 2009a). Thus, the stability of amyloid fibrils is an important physical factor that modulates their biological function of amyloid and potential as a nanomaterial.

The kinetics of the nucleated growth of amyloid fibrils are profoundly influenced by secondary processes such as fibril fragmentation/breakage (Knowles et al., 2009; Xue et al., 2008) and secondary surface nucleation (Buell et al., 2014; Tornquist et al., 2018) (Figure 1A). These processes determine the rate of the exponential growth phase of amyloid assembly alongside with growth by elongation at fibril ends (Lorenzen et al., 2012; Xue et al., 2008). As one of the key secondary processes, fibril fragmentation stands out compared with the other three main processes (Figure 1A) in that it reduces the aggregate size at the same time as it increases the number of aggregates (Xue et al., 2009a). In this aspect, fibril fragmentation results in the division of amyloid fibrils analogous to a microbial or cellular division process. Resistance to fibril fragmentation is linked to the mechanical stability of amyloid fibrils, which has implications for both the development of nanomaterials and on the understanding of amyloid disease-associated biological processes. The mechanism and the rate of fibril fragmentation have been subjected to theoretical considerations (Hill, 1983; Knowles et al., 2009; Paparcone and Buehler, 2011; Xue et al., 2008) and experimental investigations involving fragmentation promoted by mechanical perturbations (Nicoud et al., 2015; Xue et al., 2008; Xue and Radford, 2013). The fragmentation of protein filaments is a length-dependent process whereby longer particles break more easily than short ones. This length-dependent breakage of amyloid fibrils can follow a strong, non-linear dependence where longer fibrils are progressively less stable toward breakage per monomeric unit relative to their shorter counterparts (Xue and Radford, 2013). Thus, the fibrils' resistance to division, and in turn the inherent stability of the fibrils, is an important and measurable property (Xue and Radford, 2013) that will help rationalize phenomena such as prion strains, polymorphism, transmission, amyloid toxicity, biofilm formation, and epigenetic regulation (e.g., Aguzzi et al., 2007; Cox et al., 2003; Derdowski et al., 2010; Lee et al., 2011; Lin et al., 2017; Marchante et al., 2017; Shorter and Lindquist, 2004; Sondheimer and Lindquist, 2000; Tanaka et al., 2006; Xue et al., 2009a; Zeng et al., 2015) and lead to a better understanding of amyloid-associated diseases.

The division of amyloid polymers into small more infective particles, either through environmental perturbations or through catalysis by molecular chaperones, is key to the spreading of prion phenotypes (Cox et al., 2003; Marchante et al., 2017). For example, the propagation of the yeast prion phenotype [*PSI*^*+*^] associated with yeast Sup35 protein assemblies relies on the fragmentation activity of the chaperon Hsp104 and its co-chaperones (Chernoff et al., 1995; Shorter and Lindquist, 2004). The resistance of Sup35 assemblies to fragmentation correlates with the formation of different [*PSI*^*+*^] phenotypes (Tanaka et al., 2006). In addition, the smaller particles generated by fibril fragmentation show enhanced cytotoxicity when compared with the larger parent fibrils (Xue et al., 2009a), likely due to a higher propensity to interact with cell membranes, entering cells by endocytosis, interacting with the lysosome, and inducing cytotoxicity by disrupting proteostasis (Ankarcrona et al., 2016; Hu et al., 2009; Jakhria et al., 2014; Marchante et al., 2017; Milanesi et al., 2012). The stability of amyloid fibrils toward division is, therefore, an important characteristic of amyloid fibrils that must be considered if we are to understand the biological activity and nanomaterial properties of amyloid. As protein filaments formed from different precursors show a variety of suprastructures and size distributions (e.g., Barritt et al., 2017; Knowles et al., 2007; Meinhardt et al., 2009; Xue et al., 2009a), no unifying theory has been developed for the division of amyloid fibrils. As consequence, the stability toward division for different types of amyloid fibrils with varied suprastructures that ranges from inert network of long filaments to infectious particles is yet to be systematically measured, determined, and compared.

We have previously shown that the time evolution of amyloid fibril length distributions obtained by nanoscale atomic force microscopy (AFM) imaging contain valuable information on the rate, length dependence, and position dependence of fibril fragmentation that can be extracted (Xue and Radford, 2013). However, as fibril division is itself a strongly length-dependent process, systematic comparison of the stability of fibrils toward division and their division rates has been hampered by the varied length distributions of different types of amyloid fibrils. Currently, the links between data and theory that would allow direct comparison of the fibrils' division propensities are also missing. Here, we have developed an analytical approach that enables direct determination of the dynamic stability of amyloid fibrils toward division from fibril length distributions. We have developed a new theory on amyloid fibril division that shows how the division mechanism of amyloid fibrils and their stability toward division dictates the exact shape of the resulting length distributions. We then established an analytical method to extract a set of unique and intrinsic properties of the fibril stability to division from image data of pre-formed fibrils undergoing physical fragmentation experimentally promoted by mechanical perturbation. Demonstrating the utility of our combined experimental and theoretical approach, we determined and compared the division of fibril samples formed from human α-synuclein (α-Syn) associated with Parkinson disease with fibrils formed from β-lactoglobulin (β-Lac) and lysozyme (Lyz). We have also reanalyzed and compared previously published fibril fragmentation data of β_2_-microglobulin (β_2_m) under the same mechanical perturbation regime (Xue and Radford, 2013). Comparison of the dynamic stability of these fibril types of different origin revealed different division properties, with fibrils formed from the human Parkinson disease-associated α-Syn being the least overall stable and prone to generate small sub-100-nm particles that may possess enhanced cytotoxic and prion-like infectious potential (Brundin and Melki, 2017). The ability to assess and compare the division properties of amyloid fibrils, enumerated as parameters extractable from experimental data, enables the prediction of an amyloid's propensity to generate toxic and infectious particles and therefore has a significant impact on the understanding of their roles in biology, in diseases, and their application as a functional bio-nanomaterial.

## Results

### Amyloid Fibrils of Diverse Suprastructures and Length Distributions Fragment to Different Extents upon Mechanical Perturbation

**Figure 2:**
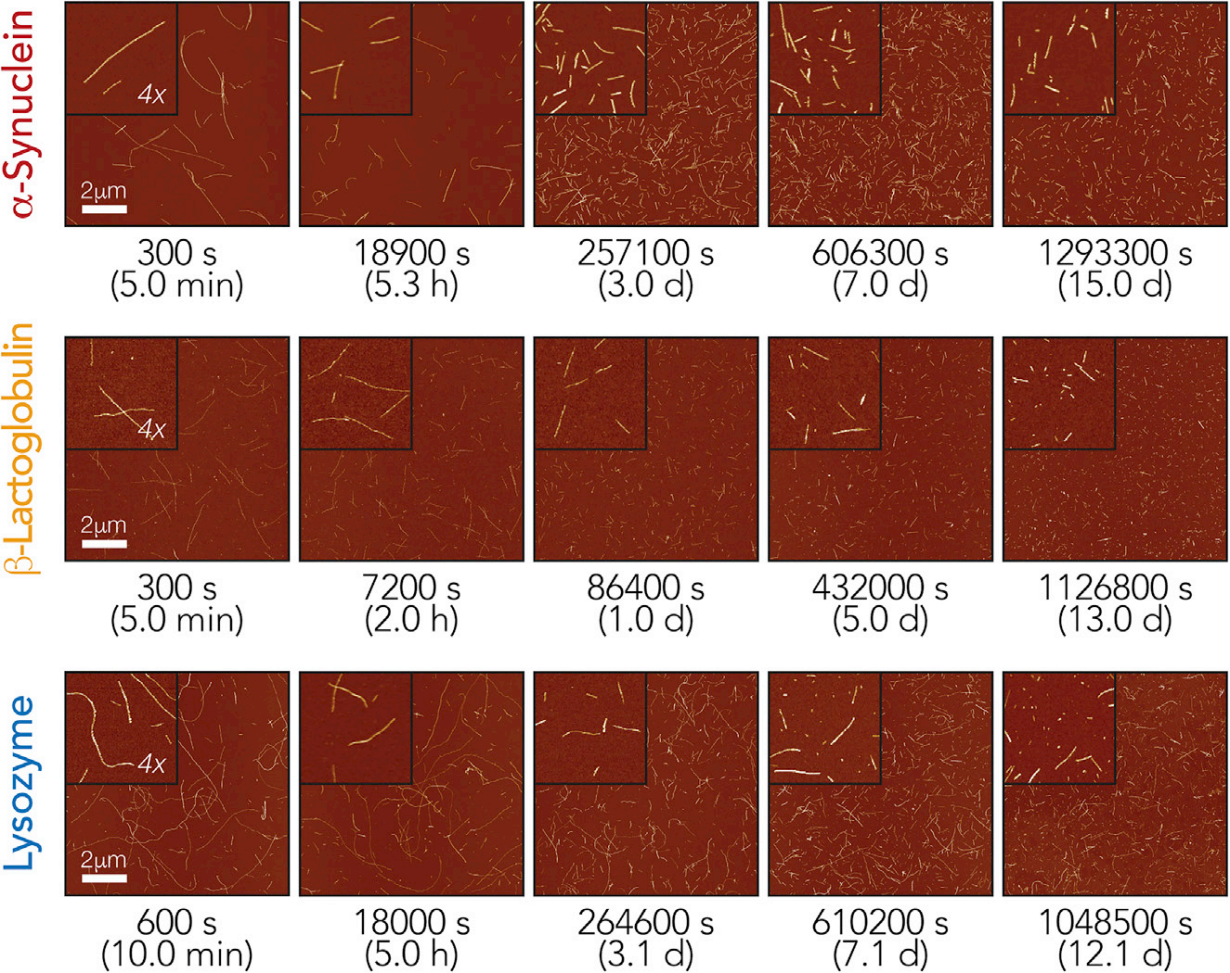
AFM Imaging of Amyloid Fibrils Undergoing Fragmentation Promoted by Mechanical Stirring Hen egg white Lyz, bovine milk β-Lac, and human α-Syn amyloid fibril samples (all 120 μM monomer equivalent concentration) were stirred for up to 15 days. Samples were taken out periodically, deposited on mica, and imaged using AFM. Typical AFM images representing 10 × 10 μm surface areas are shown together with 4× magnified insets. Scale bars, 2 μm.

To demonstrate that the fibril division rates, indicative of their dynamic stability to division, can be assessed and compared for amyloid fibrils with diverse suprastructures and length distributions, we first collected experimental AFM image datasets of amyloid fibrils, pre-formed from different precursors, undergoing division through fragmentation promoted by mechanical stirring. These experiments were designed to isolate the fibril division processes from other growth processes and to generate data that contain sufficient quality and quantity of information on the division of fibril particles under identical mechanical perturbation regimes to enable comparison. Here, we chose to investigate the human disease-associated amyloid system α-Syn alongside bovine β-Lac and chicken egg white Lyz as biophysical model systems not directly related to human disease. Samples were formed containing long, straight fibrils from these three proteins *in vitro* and validated by negative-stain electron microscopic imaging (Figure S1). Lyz and β-Lac were both converted to their fibrillar amyloid form by heating under acidic conditions (pH 2.0), commonly used conditions for the assembly of these proteins *in vitro*. α-Syn fibrils were prepared from freshly purified recombinant α-Syn monomers (Cappai et al., 2005) at 37°C under physiological pH. For each fibril sample, 500 μL of 120 μM monomer equivalent fibril solutions in their respective fibril forming buffer were then stirred at 1,000 rpm by a 3 × 8-mm magnetic stirrer bar in a 1.5-mL glass chromatography vial using the same mechanical perturbation method as previously reported (Xue and Radford, 2013) with an Ika Squid stirrer plate with a digital display. The *in vitro*-formed fibril samples were initially dispersed by 5–10 min of stirring and were subsequently deposited onto freshly cleaved mica surfaces and imaged by AFM (Figure 2 leftmost column).

As seen in the leftmost column of images in Figure 2, the initial samples after brief stirring to disperse the fibril particles show long, straight, elongated, unbranched nanostructures expected for amyloid fibrils. However, whereas Lyz and α-Syn form fibrils that exhibit more flexibility and curvature, β-Lac forms comparably shorter, straighter, more rigid assemblies consistent with previous observations (e.g., Knowles et al., 2007; Lara et al., 2011; Nicoud et al., 2015; Sweers et al., 2012). The Lyz and β-Lac images also display higher background noise compared with the images of α-Syn fibrils, which may reflect their overall less-efficient fibril assembly reaction conditions compared with α-Syn. Importantly, however, all the samples showed well-dispersed fibril particles that can be individually measured after the brief stirring treatment, as the samples did not show strong propensity for clumping on the images.

The samples were then continuously stirred for up to 15 days, and 1–5 μL samples (see Transparent Methods) were taken out periodically and imaged using AFM to visualize their fragmentation under mechanical perturbation (Figure 2). For each sampling time point, an identical AFM specimen preparation procedure was used for each amyloid type, and 20 μm × 20 μm surface areas were imaged at 2,048 × 2,048-pixel resolution to enable quantitative analysis of individual fibril particles as previously described (Xue, 2013; Xue et al., 2009b). In total, fragmentation of two independent fibril samples was followed for each fibril type, and 171 images with at least 300 particles for each sample and time point were analyzed, giving a total dataset containing physical measurements of more than 220,000 individual fibril particles for the three amyloid types (Table S1).

**Figure 3:**
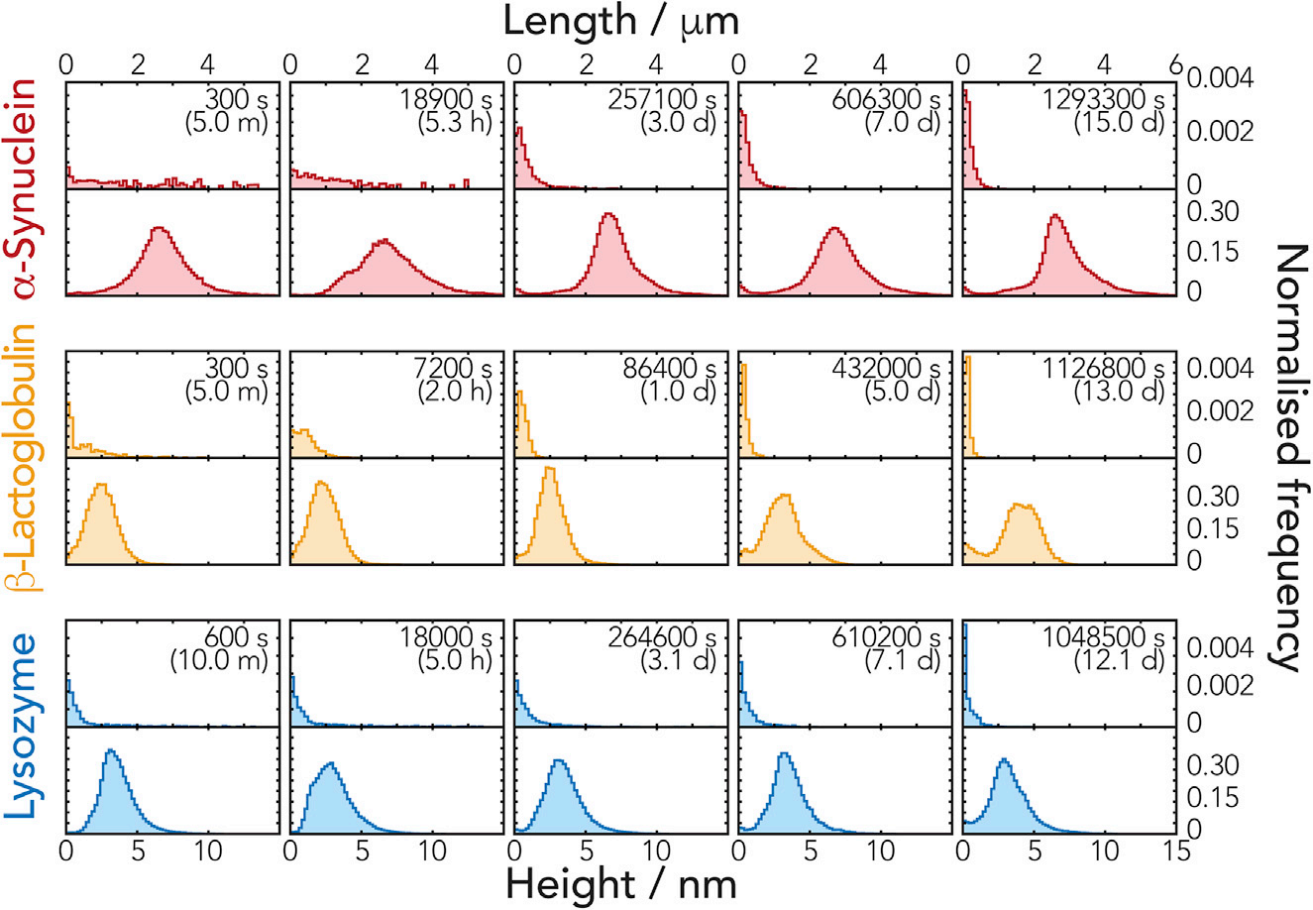
Fibril Length and Height Distributions Extracted from AFM Images of the Fibrils Undergoing Fragmentation by Mechanical Perturbation Normalized length (upper row of each sample) and height (lower row of each sample) distributions of fibril particles corresponding to the same AFM images in Figure 2 are shown as histograms. The histograms are shown using the same length and height scales, respectively, for comparison.

Quantitative single-particle measurements of fibril length and height distributions (Figure 3, leftmost column corresponding to images in Figure 2 leftmost column) reveal that the fibrils have substantially different initial dimensions. Analysis of their height distributions shows that the initial fibril heights, indicative of the width of the fibrils, are around 7 nm for α-Syn fibrils and around 3 nm for both β-Lac and Lyz fibrils. The initial length distributions for the different fibril types were also dissimilar, with both Lyz and α-Syn forming fibrils of up to ∼10 μm in length, whereas β-Lac formed shorter particles with lengths of up to ∼2 μm under the conditions employed.

Qualitative inspection of the AFM images throughout the experiment (Figure 3) showed that the amyloid fibrils were fragmented into much smaller particles under the applied mechanical perturbation (Figures 2 and 3) as expected. However, the rate of division and shortening of the particles' lengths was seen to differ between the three different fibril types analyzed (Figures 3 and S2). Analysis of the time evolution of the fibril height and length distributions obtained by quantification of individual particles in the AFM images over the course of the experiment confirmed that fibril fragmentation did not cause detectable changes in fibril morphology and fibril width through lateral association and dissociation. Average fibril heights in the AFM images, indicative of fibril widths, remained consistent throughout the experiment for Lyz and α-Syn. The same was also largely observed for β-Lac, with the exception that a small second population of taller polymers at the very end of the fragmentation time course after 432,000 s was observed (height graphs in Figures 3 and S2). Hence the division of the fibrils under mechanical perturbation applied has resulted in a shortening of average fibril length.

To confirm that the changes in fibril length by fibril division did not cause disaggregation or release of monomer/small oligomers (e.g., dimers), we next determined the residual monomer concentration of the samples. For each fibril type, aggregates were pelleted by centrifugation (75k rpm, 15 min) after fragmentation time course and the presence of monomer in the supernatants was quantified by SDS-PAGE. The comparison between the initial samples and those fragmented over 2 weeks showed no substantial changes in the protein composition of the supernatants, with differences of less than 2% for all amyloid systems analyzed (Lyz: 1.4%, β-Lac: <1%, and α-Syn: 1.3%, Figure S3). These data confirmed that the time-dependent imaging experiments we carried out pertain almost exclusively to the fibril division processes along the length of the pre-formed fibrils and therefore contain valuable information on their division rates and their stability to division.

### Time Evolution of Fibril Length Distributions Converges to Time-Independent, Characteristic, Self-Similar Length Distribution Shapes

The fibril samples formed from different protein precursors have different initial length distributions (as seen in Figures 2 and 3). However, fibril division is itself a strongly length-dependent process (Xue and Radford, 2013) as short fibril particles will be more resistant to division compared with longer particles, irrespective of any differences in the intrinsic stability of the different fibril types to division. Therefore, to compare the stability of amyloid fibrils with different suprastructures and length distributions toward division, a new approach to extract information intrinsic to each fibril type independent of their experimentally different initial length distributions must be developed. Consequently, in parallel with the experiments described earlier in the article, we mathematically analyzed the division equation of amyloid fibrils so that key information on the stability of amyloid fibrils to division could be resolved. We first describe the division of amyloid fibrils mathematically using a continuous framework based on the partial differential equation (PDE) Equation 1. As the number of monomers inside a fibril observed in the image data is large, typically in the order of 10^2^ or more, we assumed continuous variables *x* and *y* that correspond to the length of fibrils (for example, as defined in Figure 1B where y is the length of the parent fibril and x is the length of one of the daughter fibrils). This approach has the advantage that the infinite set of ordinary differential equations (ODEs) normally used to describe the length-dependent division processes (e.g., Knowles et al., 2009; Xue et al., 2008; Xue and Radford, 2013) can now be collapsed into a single continuous PDE that can be treated analytically (see Supplemental Information for details). Denoting *u(t, x)* as the distribution of fibrils of length *x* at time *t* in number concentration units (e.g., molar units), Equation 1 is the mathematical translation of the pure division model described by the schematics in Figures 1B–1D, where we assume that any parent fibril can divide into two daughters and the end-to-end reattachment rate of daughter fibrils is negligible (Hill, 1983):

(Equation 1) $$\frac{\partial }{\partial t}u\left(t,\;x\right)=\;-\alpha_{0}{\left(\alpha x\right)}^{\gamma }u\left(t,\;x\right)+\;2\int_{y=x}^{\infty }\frac{1}{y}\kappa_{0}\left(\frac{x}{y}\right)\alpha_{0}{\left(\alpha x\right)}^{\gamma }u\left(t,\;y\right)dy$$

**Figure 4:**
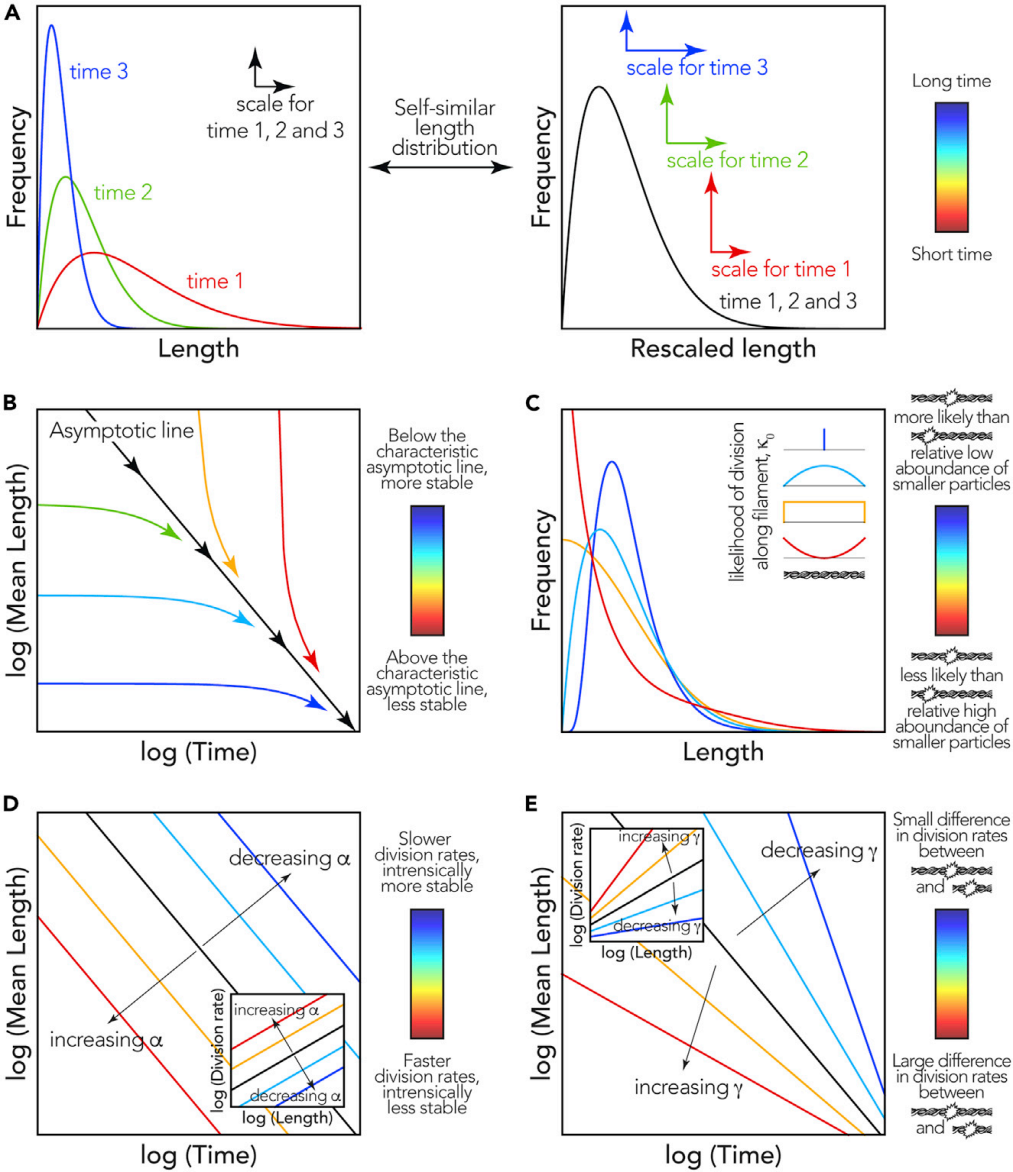
Illustration of The Key Insights Emerging from the Mathematical Analysis of the Division Model The behavior of the division equation Equation 1 is entirely and uniquely dictated by a set of three properties: α, γ, and κ_0_. Several key predictive insights emerged from the analytical solution of Equation 1 with regard to these three properties. (A) The three example length distributions in the left panel can be rescaled to show the same distribution shape in the right panel, illustrating the concept of self-similar length distributions. (B) After a period of time, the self-similar length distribution shape is reached. From this point, the reduction in the average length of the fibril length distribution can be described as a power law versus time. The decay of mean length of a sample is predicted to tend toward a straight line, the asymptotic line, when plotted on a log-log plot with the slope of the line representing −1/γ (black line in B, D, and E). The asymptotic line denoting mean fibril lengths decay also does not depend on the initial length distribution (colored lines in B). (C) The self-similar length distribution shape contains information about κ_0_, which describes how likely a fibril will divide in the middle versus shedding a small fragment from the edge. A κ_0_ indicative of fibril types that are more likely to divide in the middle will result in fibril length distributions with a distinct peak and low relative population of small fragments (dark blue and light blue curves). In contrast, κ_0_ indicative of fibril types and conditions that promote equal likelihood of division along the fibril or even favor shedding of small fragments from fibril edges will result in self-similar fibril length distributions that have a larger relative population of small fibril fragments (orange and red curves) compared with κ_0_ values favoring division in the center of the fibrils. (D and E) (D) and (E) illustrate how the black asymptotic line describing the decay of fibril lengths in (A) is dictated by the parameters α and γ, respectively. For each panel, the color bar to the right illustrates the different properties associated with the colors in the panel (e.g., division in the center versus at the edge of a fibril for (C), and division of a long versus a short fibril in (E)).

In Equation 1, *∂u(t, x)/∂t* denotes the time (*t*) evolution of the concentration of fibrils with length *x*. Here, we model the total division rate constant of fibrils of size *x* using the power law *α*_0_(*αx*)^*γ*^ (Hill, 1983), which we denote as *B*(*x*) (see Supplemental Information), where *α*_0_ is a constant unit reference we set to 1 s^−1^. The first term in Equation 1 therefore, denotes the rate of loss of fibrils with length *x* by division into smaller fibrils. The probability that after dividing, a given parent fibril of length *y* gives rise to daughter fibril fragments of length *x* and *y-x* depends on the ratio of the lengths (*x/y*) (Xue and Radford, 2013) and is given by the probability density function (1/*y*)*κ*_0_(*x*/*y*). The second integral term in Equation 1, therefore, denotes the total gain of fibrils with length *x* by division of all fibrils with length *y* that are larger than *x*. Interestingly, Equation 1 describes a fundamental division process that is mathematically analogous to the division of molecules, macroscopic materials, and cells (Escobedo et al., 2005; Robert et al., 2014), and we have mathematically proved that its behavior is entirely and uniquely dictated by three properties: *α* that describes the magnitude of the division rate constant, *γ* that describes the fibril length dependence of the division rate constant, and *κ*_*0*_ that describes the probability of division at any given position along a fibril, also called the fragmentation kernel (Doumic et al., 2018). We then proceeded to solve Equation 1 analytically with regard to *α, γ,* and *κ*_*0*_ using theoretical results shown in Escobedo et al. (2005) and Doumic et al. (2018) (see Supplemental Information). From our solution, we note four key predictive insights that emerged from our analysis (Figure 4).

First, we note that given enough time, the decay of the average fibril length will converge to the same rate independently of the initial fibril length distributions. This result comes from that after a sufficiently long time, the reduction of average length of the fibril length distribution can be described as a power law versus time (Equation 2, see Supplemental Information):

(Equation 2) $$\mu \left(t\right)\;=\;C\cdot t^{-1/\gamma }$$

where *C* is a constant. As seen in Equation 2, the experimentally observable average length of a sample, *μ*(*t*), is predicted to tend toward a straight line when plotted on a log-log plot with the slope of the line representing −1/γ (Equation 2, black line in Figure 4B) because the long-time behavior of Equation 1 can be described as a power law.

Second, we note that given enough time, the fibril length distribution will converge to the same shape independently of the initial state of the fibril length distribution. After a sufficiently long time (*t*≫*t*_0_), the distribution of fibril lengths tends toward a time-independent distribution shape, *g*(*x*_*g*_), that scales only with *t* and γ, but does not depend on the initial length distribution (Equation 3 and Supplemental Information).

(Equation 3) $$g\left(x_{g}\right)\approx f\left(t,x\right)\cdot t^{-\frac{1}{\gamma }},\;\;x_{g}=\;xt^{\frac{1}{\gamma }},\;\;For\;any\;t\gg t_{0}$$

where *f(t, x)* are experimentally measured length distributions. This point is of key importance for characterizing and predicting fibril division processes because it establishes that for any fibril type under certain conditions (1) a distinct fibril length distribution shape (Figure 4A) will be reached independently of the initial fibril length distribution and (2) the length distribution and the average length will shrink as function of time in a predictive manner as fibrils continue to divide (e.g., the black line in Figure 4B for the mean length) but the shape of the distribution will not change as function of time, i.e., the length distribution can be rescaled to the same *g*(*x*_*g*_) using Equation 3 at any time *t* along the black line in Figure 4B if *t* is sufficiently large. We refer to the distributions with the scaling property and shape invariance property as “self-similar length distributions” (Figure 4A).

The existence of a self-similar length distribution that is initial length distribution independent and shape invariant over time, as well as the predictable decay of fibril lengths as fibrils divide (e.g., the reduction of the average length in Figure 4B) can be seen as a characteristic behavior specific to individual fibril types under distinct conditions. This fibril division behavior can, therefore, be classed as a type of intrinsic dynamic stability of the fibrils. One way to visualize this property is shown in Figure 4B represented by the black line, here referred to as the fibril type's “asymptotic line” under the conditions applied. Any fibril population above this line is relatively unstable and will rapidly divide, pushing the average length toward the line (red- and yellow-colored near-vertical arrows showing rapid decay of unstable fibril lengths). In contrast, any fibril population below this line is comparatively stable or metastable and will only slowly evolve toward the line through division (green- to blue-colored near-horizontal arrows showing slow decay of stable fibril lengths toward the black line). Importantly, this result also indicates that the dynamic stability of fibrils to division represented by the asymptotic line (1) can be determined from experimental data, (2) is intrinsic to fibril type and conditions applied, and (3) can be compared independently of varied starting fibril length distributions, if the characteristic self-similar length distributions that contain information about the intrinsic dynamic stability of the fibrils is reached (e.g., the asymptotic line is reached in an experiment running for sufficiently long length of time).

Third, we note that the probability of division at the center of a fibril when compared with the shedding of small particles from fibril edge can be evaluated from the experiments. The self-similar length distributions contain information about κ_0_. Figure 4C shows how different self-similar fibril length distributions are indicative of different κ_0_ probability functions. As seen in Figure 4C, a κ_0_ indicative of fibril types that are more likely to divide in the middle will result in fibril length distributions with a distinct peak and low relative population of small fragments. In contrast, a κ_0_ indicative of fibril types and conditions that promote equal likelihood of division along the fibril or even favoring the shedding of fragments from fibril edges will result in self-similar fibril length distributions that have large relative population of small fibril fragments that may possess enhanced cytotoxic and/or infective potential compared with κ_0_ favoring division in the center of the fibrils.

Finally, the dynamic stability of fibrils to division, their propensity to break at different lengths, can be determined. The first-order division rate constant *B*(*x*) = *α*_0_(*αx*)^*γ*^ that describes the division of the fibrils as a function of their length *x* can be directly evaluated from the self-similar length distribution and γ (see Equation 2) when *t >> t*_*0*_ (see Supplemental Information and Equation S21) where *t*_*0*_ is the start of the experiment. Thus, the division rate constant *B(x)* can be determined by experimentally observing how fibril length distributions change with time when the self-similar fibril length distribution is obtained, and they are important parameters for defining and comparing the fibrils' intrinsic dynamic stability to division. The effect of different values of *α* and *γ* on fibril stability is visualized in Figures 4D and 4E as characteristic of the asymptotic line plotted in log-log plots of average length versus time. The enumeration of the asymptotic line described by *B(x)* will subsequently enable direct quantitative comparison of the fibrils' stabilities toward division.

### The Division Properties of Amyloid Fibrils Can Be Obtained from Image Data, and Their Complex Stability to Division Can Be Compared

**Figure 5:**
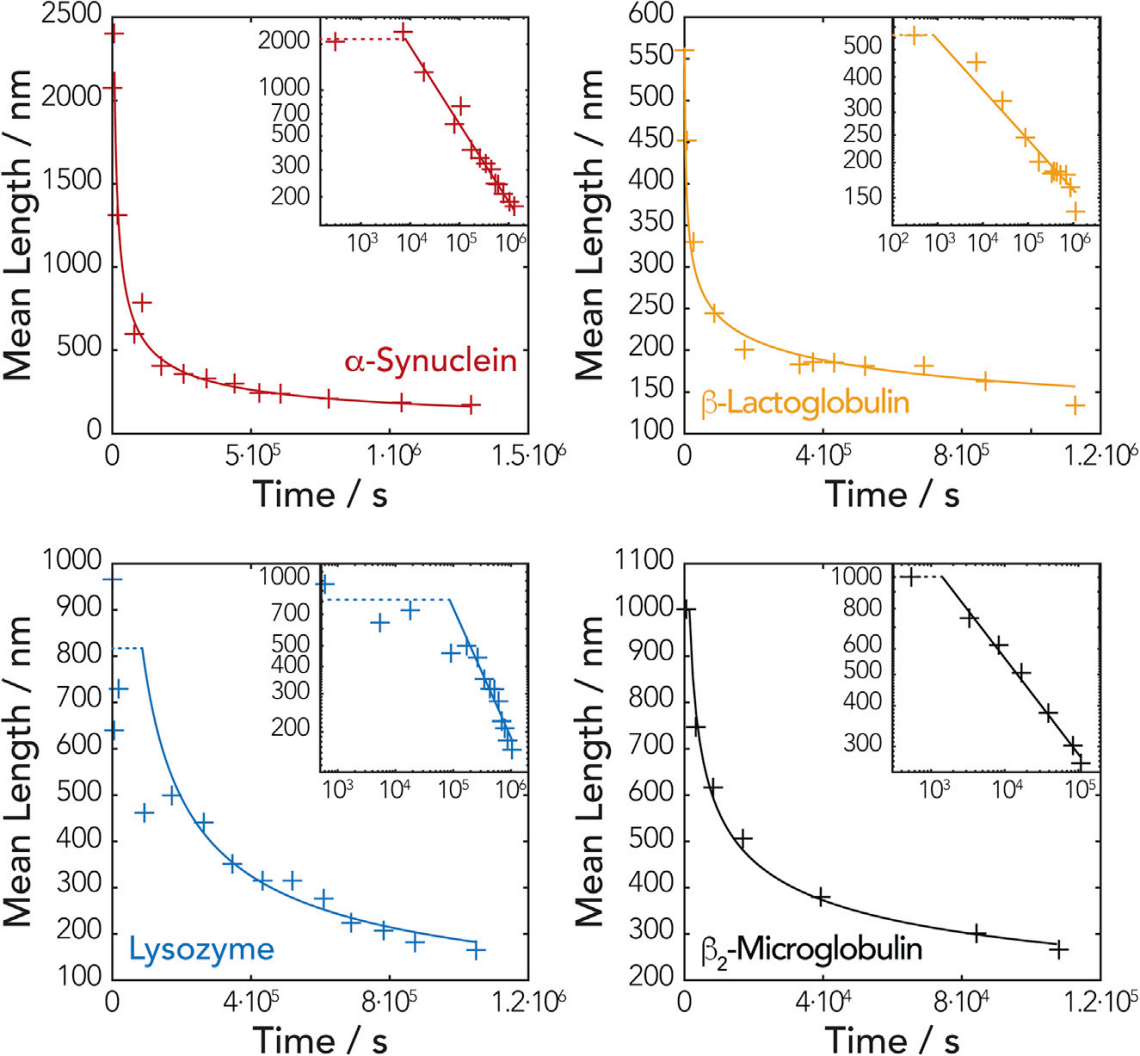
Fitting the Fibril Division Model to Fibril Length Decay Data Extracted from AFM Images The analytical solution of our division model shows the decay of average length as function of the γ parameter in Equations 2 and S22. Equation S22 was fitted to the decay of average fibril length during division for each of the fibril types analyzed (including previously published data for β_2_m fragmentation under the same mechanical perturbation conditions in Xue and Radford (2013). The solid fitted lines represent the time regimen where the length distributions closely approached the asymptotic line and the self-similar distribution shape where Equation 2 is valid (Transparent Methods).

Applying the results of the mathematical analysis to the experimental AFM image datasets, the parameters *γ*, *α*, and the characteristic self-similar length-distributions *g*(*x*_*g*_) indicative of *κ*_*0*_ can be extracted and meaningfully compared as a measure of the fibrils' intrinsic stability to division. We first determined the *γ* values for each of the fibril types, by globally fitting a variant of Equation 2 to the time evolution of average fibril length (see Transparent Methods, Figure 5). We also reanalyzed previously published dataset on β_2_m fibril fragmentation under the same mechanical perturbation conditions (Xue and Radford, 2013) using our new aforementioned theoretical results and included the reanalysis in the comparison.

The constant *γ* was determined from least-squares fitting of our analytical result to the data (Transparent Methods). The power law relationship (Equation 2) parameterized with *γ* determined by global analysis was visualized on a log-log plot of mean fibril length versus time in Figure 5, together with the measured mean fibril lengths. The resulting *γ* values are listed in Table 1. A *γ* value of 1 would suggest that the division rate of fibrils is only dependent on the number of division sites per fibril, which is linearly related to the number of monomers in the fibrils and in turn to the length of the fibrils. However, the *γ* values for α-Syn, β-Lac, and β_2_m are all significantly larger than 1, indicating highly length-dependent microscopic division rates for division sites in these fibril types. Of the four fibril types analyzed, the division of Lyz fibrils yielded a *γ* value closest to 1. This suggests that the division rates for Lyz fibrils may only depend on the number of available division sites along the fibrils. β-Lac fibrils yielded the highest *γ* value of the fibril types analyzed. This demonstrates that β-Lac fibril fragmentation is highly length dependent, and small β-Lac fibril fragments are more resistant to further fragmentation compared with the other fibril types. This behavior may corroborate with an increased lateral association of small β-Lac fibril fragmentation fragments observed on the height distributions at the end of the time course experiments (height graphs in Figure 3 and S2). As seen in Figure 5, the later time points for all our fibril types follow a straight line on the log-log plots (solid section of the fitted lines in Figure 5), indicating that the self-similar length distributions, and hence the asymptotic line, were sufficiently reached in all cases. The analysis also revealed that all the fibril types analyzed approached the self-similar length distribution shapes in less than 5 h, with the exception of the Lyz samples that reached the self-similar distribution in approximately 24 h.

**Table 1:** Parameters from the Division Analysis of the Different Fibril Types

*Sample*	*γ ± SE*	*α/nm*^*−1*^*(log α ± SE)*	*B (100 nm)/s*^*−1*^*(log B ± SE)*	*Height (Fibril Width)/nm*
*α-Syn*	2.0 ± 0.3	2.6 × 10^−6^ (−5.6 ± 0.2)	9.2 × 10^−8^ (−7.0 ± 0.3)	6.8 ± 0.6
*β-Lac*	5.7 ± 0.8	1.8 × 10^−4^ (−3.7 ± 0.2)	1.2 × 10^−10^ (−9.9 ± 0.8)	3.0 ± 0.5
*Lyz*	1.7 ± 1.0	9.4 × 10^−7^ (−6.0 ± 0.9)	2.0 × 10^−7^ (−6.7 ± 1.0)	3.1 ± 0.4
*β*_*2*_*m*^a^	3.4 ± 0.4	5.6 × 10^−5^ (−4.3 ± 0.3)	2.5 × 10^−8^ (−7.6 ± 0.4)	5.4 ± 0.6

a Reanalysis of data from Xue and Radford (2013).

**Figure 6:**
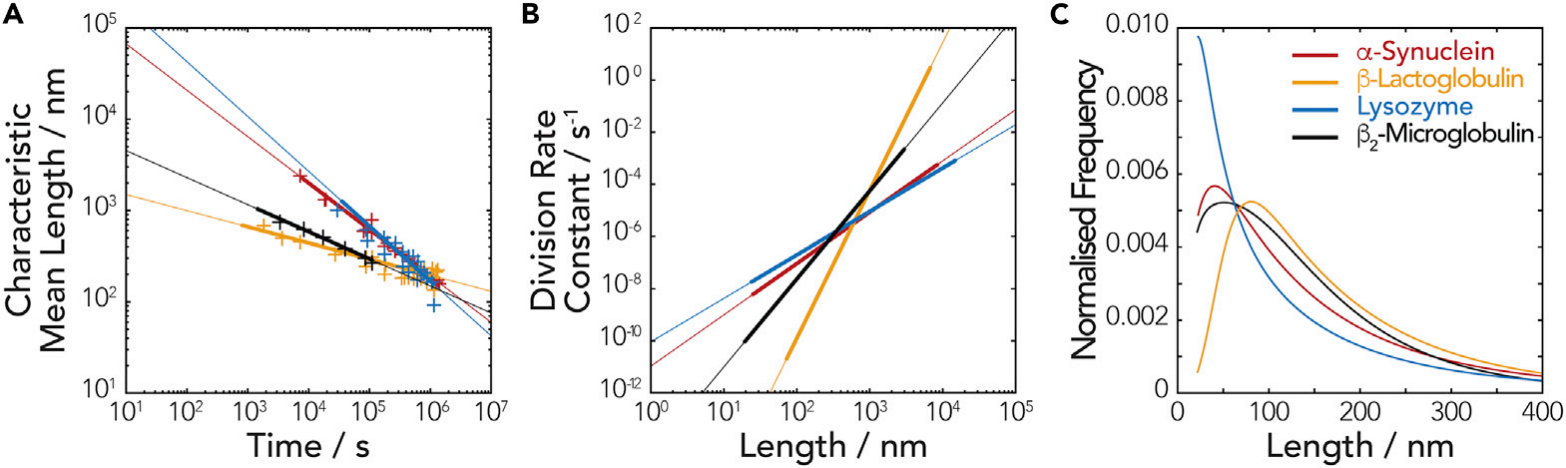
Comparing the Stability toward Division Of Different Amyloid Fibril Types (A–C) The decay of mean lengths (A), the division rate constants as function of fibril length (B), and the self-similar length distribution shapes (C) for hen egg Lyz (blue), bovine milk β-Lac (yellow), human α-Syn (red), and human β_2_m (black; data from Xue and Radford, 2013) amyloid fibril samples undergoing division by fibril fragmentation under mechanical perturbation. All curves were calculated using α, γ, and g(x_g_) obtained from our analysis of the experimental AFM images. In (A), the thicker portion of the lines denote the time range where the characteristic self-similar length distribution shape is observed in the imaging experiments (i.e., corresponding to the time regime represented by the solid fitted lines in Figure 5), and crosses are the experimental data points that have closely reached the self-similar distribution shapes shown in the same plot. In (B), the thicker portion of the lines denotes the range of fibril lengths observed experimentally on the AFM images. In (C), the distributions were calculated using self-similar distributions g(x_g_) in Figure S3 after 2 weeks.

The *α* values were subsequently calculated (listed in Table 1) with Equation S21 using all the fibril length distributions at time points post reaching the near-characteristic self-similar distribution shapes (represented by the solid lines in Figure 5). Once both *α* and *γ* values have been extracted from the length distribution data, the division rate constant *B(x)* can be obtained for fibrils of any length *x*. Table 1 shows the division rate constant calculated for fibrils of 100 nm. The asymptotic line for the fibrils types characterized by the division rate constant *B(x)* (Figure 6B) or by fibril mean length (Figure 6A) as a function of time was also visualized and compared independently of initial fibril length, showing that α-Syn and Lyz fibrils fragment the fastest at long times under the mechanical perturbation applied, suggesting that these fibrils were less stable than the β-Lac and β_2_m fibrils.

Next, we determined the shape of the self-similar length distributions for each fibril type by rescaling the experimental length distributions to *g*(*x*_*g*_) with Equation 3 using the γ values obtained earlier. As with the evaluation of *α* values, only time points where the length distributions closely approached the self-similar length distribution (time points in the section represented by the solid lines in Figure 5) were averaged to obtain *g*(*x*_*g*_) for each fibril type (Figure S4). Figure 6C shows how the self-similar length distribution shapes compare with each other at extended times (2 weeks) when calculated using *g*(*x*_*g*_) (Figure S4) with Equation 3. As seen in Figure 6C, Lyz fibrils tend to produce high relative populations of small particles less than 100 nm long followed by α-Syn and then β_2_m. On the other hand, the division of β-Lac fibrils resulted in a lower relative population of small particles over the same long timescale used for the other fibril types.

**Figure 7:**
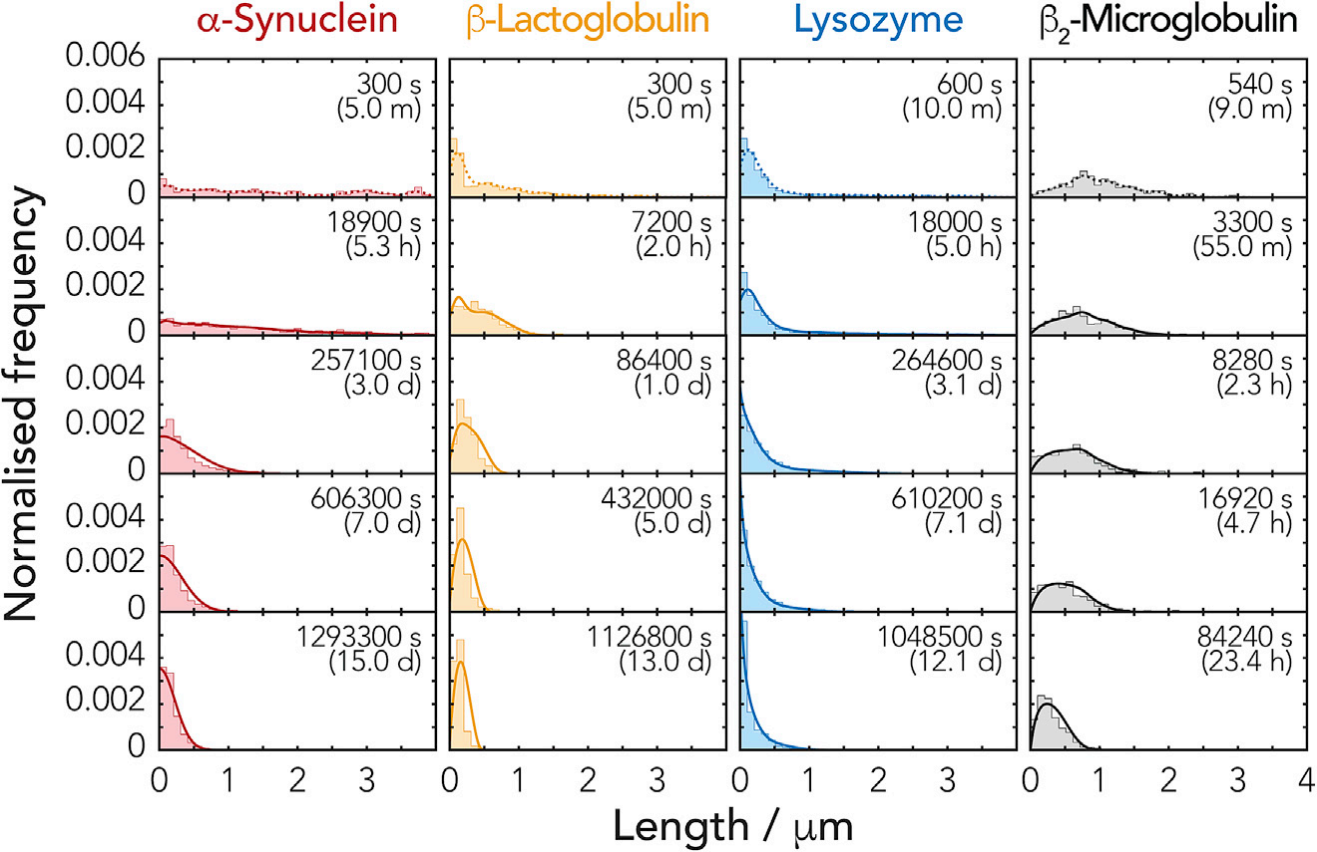
Validation of the Division Parameters α, γ, and κ_0_ and Their Predictive Power Full direct simulation of fibril fragmentation processes was carried out using α, γ, and κ_0_ determined from the image data. For each fibril type, the initial normalized frequency distribution (dashed lines in top row) was used directly as the initial state for the simulations. The resulting simulated evolution of length distributions solely based on the calculated α and γ values and estimated shapes κ_0_ (see Methods) are compared with the experimental data shown as histograms.

Finally, to validate our model and the predictive power of our approach, we performed direct simulations of the fibril division time course (Figure 7) using only the individual sets of division parameters obtained for each of our fibril types. For each simulation, we used the initial experimental length distributions (dashed lines in Figure 7) directly as the starting points for the simulations. The large set of ordinary differential equations describing the chemical master equation for the system (Xue and Radford, 2013) was then solved to see whether our analytical model was able to predict the full division behavior and the time evolution of the fibril length distributions for each fibril type. As seen in Figure 7, the result of the numerical simulations based on our results show remarkable agreement with the experimental data. This unequivocal result validated the fact that the set of three properties *γ*, *α*, and *κ*_*0*_ are indeed capable of fully and uniquely describing the complex amyloid division processes, and the enumeration of these properties yield valuable insights. Such insights allow meaningful comparison of the amyloid fibrils' intrinsic stability to division.

## Discussion

The understanding of the properties that underline the biological activities of amyloid nanostructures, such as their cytotoxic and infectious potentials, is crucial for understanding why some amyloids are associated with devastating human diseases. The division of amyloid fibrils, for example, through fibril fragmentation by mechanical perturbation (Xue et al., 2008; Xue and Radford, 2013), enzymatic action (Chernoff et al., 1995; Glover and Lindquist, 1998), or other cellular or environmental perturbations, is a key step in their life cycle that results in the exponential growth in the number of amyloid particles. Simultaneously, daughter particles resulting from the division of parent fibrils cause a reduction in the overall size distribution as division proceeds. These two consequences of division are undoubtedly linked to the enhancement of the cytotoxic and infectious potentials of disease-associated amyloid (Marchante et al., 2017; Xue et al., 2009a). The amyloid fibrils' resistance to division, i.e., the stability of the amyloid fibrils to division, rationalizes these two fundamental requirements for pathogenicity associated with amyloid. Akin to uncontrolled division of cells or any pathogenic microorganisms, the division step in the amyloid life cycle (Figure 1) could be a key determinant in their overall potential to be associated with properties in the amyloid and prion-associated pathology.

**Figure 8:**
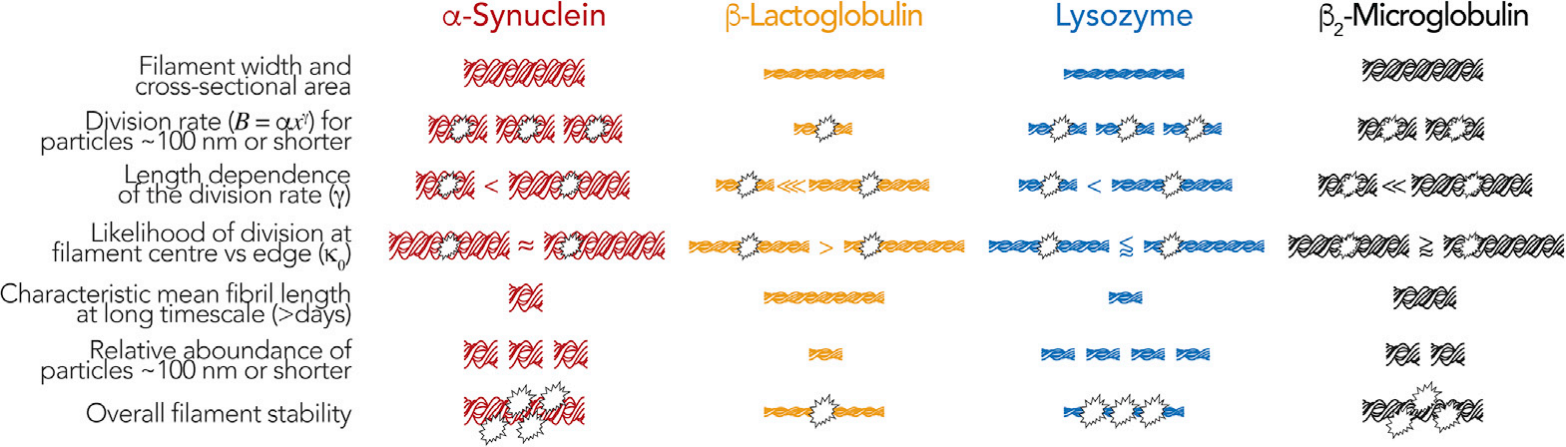
Schematic Summary of the Fibril Division Properties and Their Consequences Compared between Each of the Fibril Types Comparison of the fibril division profiles reveals differences in the dynamical stability toward breakage for the four different types of amyloid fibrils and suggests that disease-related amyloid has lowered stability toward breakage and increased likelihood of shedding smaller particles compared with amyloid not related to disease. In the illustrations, the fibril width, number, and number of breakage symbols are not to scale and denote the relative rankings for the different properties.

Here, we have developed a theory, as well as an experimental approach utilizing our theoretical insights, to resolve the amyloid fibrils' dynamic stability to division. These represent a step forward in how we are able to study the amyloid fibril division processes such as in fibril fragmentation and prion propagation, essentially the replication step in the amyloid life cycle. It also allows the direct comparison between amyloid particles of different molecular types and quantifies the difference in division and stability between those that are and are not disease associated. Specifically, we have applied our theoretical results to the comparison of a diverse set of amyloid assemblies consisting of human α-Syn (a neurodegenerative disease-associated amyloid, sample formed under physiological solution conditions), human β_2_m (a systemic amyloidosis disease-associated amyloid, sample formed under acidic pH, data from Xue and Radford, 2013), bovine β-Lac, and hen egg white Lyz (later two cases are both biophysical model systems not directly related to human disease but converted to amyloid when subjected to heating in acidic pH). By fully analyzing and comparing their division behavior, which is uniquely described by the triplet of parameters (*α,* magnitude of the division rate constant; *γ*, fibril length dependence of the division rate constant; and *κ*_*0*_, probability of division at any given position along a fibril) under identical mechanical perturbation for long timescales using our approach, we show a remarkable difference in the stability of these different amyloid assemblies relative to each other and how they divide (summarized in Figure 8). Interestingly, for the four fibril types we included here, considering the division rate constant B with their cross-sectional area, the disease-associated human α-Syn fibrils demonstrate the lowest overall stability to division followed by Lyz, human β_2_m, and finally β-Lac particles, which are the most stable toward division (Figure 8 last row). Based on the comparison of the *α* and *γ* parameters that together describe the division rates *B(x)*, the likelihood that small α-Syn particles (<100 nm long) will divide is similar to that of Lyz particles of identical length despite having more than double the mean width (and thus around four times bigger cross-sectional area, Table 1 and Figure 8). More importantly, the division of α-Syn particles also results in a larger relative concentration of small particles compared with β_2_m and β-Lac. These results show that human α-Syn amyloid fibrils are relatively unstable assemblies capable of a more rapid shedding of small particles that could well possess enhanced cytotoxic and infectious potentials (Peelaerts et al., 2015) through division compared with the other fibril types investigated here. Thus, our results also directly suggest a testable causality link between the low stability of α-Syn fibrils to division and recent observations that human α-Syn may behave in a prion-like manner in cell-to-cell propagation and their cytotoxicity (Steiner et al., 2018).

As the division of amyloid fibrils is an integral part in the propagation of the amyloid conformation (Figure 1), the nanoscale material properties of amyloid underpin processes that drive the proliferation of amyloid, as well as their varied roles in biology. Therefore, it is important to appreciate the suprastructural properties of amyloid (e.g., clustering, bundling, twist, stiffness, width distribution, orientation distribution, and length distribution, etc.) at mesoscopic (nanometre to micrometre) length scales, as these properties will influence how individual amyloid fibrils divide. Our data show that despite all amyloid consisting of a cross-beta core structure, their ability to resist division through fragmentation promoted by mechanical perturbation varies strongly between fibril types. As the stability of amyloid fibrils to division will depend on their suprastructural properties, which in turn depend on their precise structure at atomic level, mesoscopic-level structural properties may well be the missing link between amyloid structure and the varied biological effects and consequences that different amyloid types evoke under different conditions. Although the results reported here reveal the breakage behavior of fibril populations, future advances in AFM imaging may allow either individual polymorphs in a fibril population to be distinguished or individual fibrils to be tracked in real time, further revealing how fibrils divide as individuals. Thus, it should be possible to generate a structure-activity relationship correlating the suprastrucutral properties of amyloid, their ability to divide, and their cytotoxic and/or infectious potentials. Understanding this structure-activity relationship for amyloid assemblies could lead to the design of bio-safe polymers with tuned mechanical and nanomaterials properties as well as rationalize the disease-associated properties of amyloid structures.

Analogous to the diverse response of soluble folded proteins toward unfolding by chemical denaturants, thermal melting, mechanical force, etc., the stability of amyloid fibrils could also vary depending on the nature of the perturbation. Indeed, amyloid fibrils may break down in the presence of chemical, thermal, or enzymatic action (Baldwin et al., 2011; Chernoff et al., 1995; Glover and Lindquist, 1998; Knowles et al., 2007; Shammas et al., 2011; Surmacz-Chwedoruk et al., 2014), and their relative resistance or stability to different stresses, including those associated with physiological changes involved in human disorders, is not known. In particular, understanding how enzymatic action by molecular chaperones such as Hsp104 or ClpB promotes amyloid division, degradation, and/or propagation of amyloid conformation (Chernova et al., 2017; Scior et al., 2016) in relevant cases may be key in resolving the complex behavior of the amyloid life cycle in a biological context. In summary, the combined theoretical and experimental work we report here will enable the characterization and comparison of the amyloid division processes and the relative stabilities of amyloid assemblies. Both properties are fundamental in understanding the life cycle of disease-associated amyloid as well as the normal roles of functional amyloid in biology.

### Limitations of the Study

The division model (assumptions illustrated in Figure 1) does not take into account the possibilities that newly created fibril ends by division may be more dynamic, disordered, and/or be “sticky ends” in their interactions with other fibril ends or surfaces compared with established fibril ends for elongation. The results reported here reveal the overall breakage behavior of the fibril populations, as our experiments may contain a mixture of similar but, nevertheless, different polymorphs that could not be readily distinguished in our images. Future advances in AFM imaging allowing either individual polymorphs in a fibril population to be distinguished or individual fibrils to be tracked in real time will resolve breakage behavior of individual fibril polymorphs. The model assumptions and limitations may also leave scope for improvements in the model to be pursued in future work by the field.

### Resource Availability

#### Lead Contact

Wei-Feng Xue (w.f.xue@kent.ac.uk).

#### Materials Availability

This study did not generate new unique reagents.

#### Data and Code Availability

The published article includes all datasets generated and analyzed during this study. The list of all (over 220,000) raw fibril lengths and associated analysis code supporting the current study is available from the corresponding author on request.

## Methods

All methods can be found in the accompanying Transparent Methods supplemental file.

## Supplemental Information

Document S1. Transparent Methods, Figures S1–S4, and Table S1

## Footnotes

Supplemental Information can be found online at https://doi.org/10.1016/j.isci.2020.101512.
