# Supplementary material for: The Division of Amyloid Fibrils: Systematic Comparison of Fibril Fragmentation Stability by Linking Theory with Experiments
Source: iScience. 2020 Aug 29;23(9):101512. doi: 10.1016/j.isci.2020.101512 (PMC7492994; doi:10.1016/j.isci.2020.101512)
Supplement: Document S1. Transparent Methods, Figures S1–S4, and Table S1 [file mmc1.pdf]

iScience, Volume *23*

Supplemental Information

The Division of Amyloid Fibrils: Systematic Comparison of Fibril Fragmentation Stability by Linking Theory with Experiments

David M. Beal, Magali Tournus, Ricardo Marchante, Tracey J. Purton, David P. Smith, Mick F. Tuite, Marie Doumic, and Wei-Feng Xue

SUPPLEMENTARY FIGURES

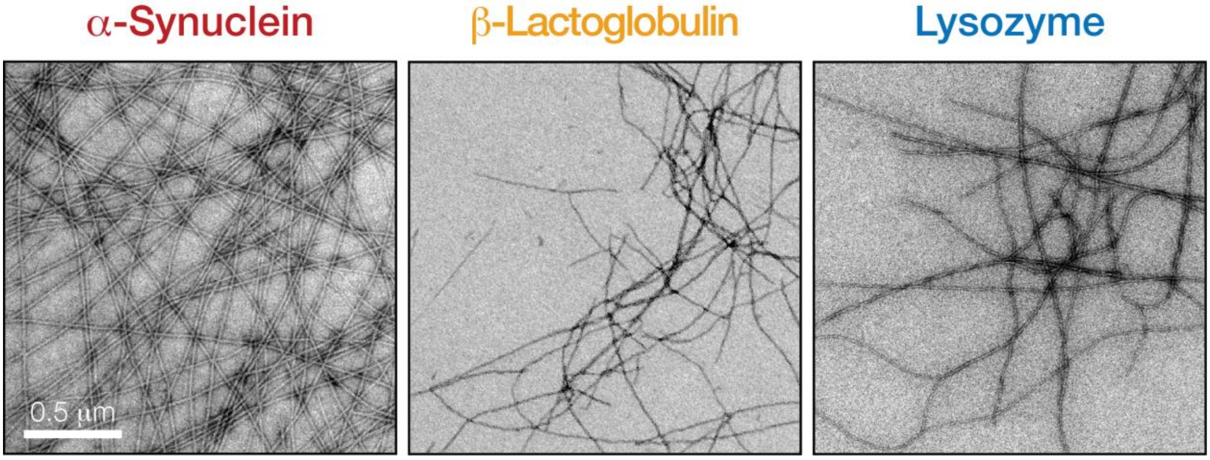

Supplementary Figure S1. Negative-stain TEM validation of the initial pre-formed amyloid fibril samples. Related to Figure 2. Initial human α-Syn, bovine milk β-Lac, and hen egg white Lyz amyloid fibril samples (all 120 μM monomer equivalent concentration) were deposited on glow-discharged, carbon coated Formvar copper grids and imaged using TEM after staining with 2% (w/v) uranyl acetate. Typical TEM images are shown with scale bar represents 500 nm in all images.

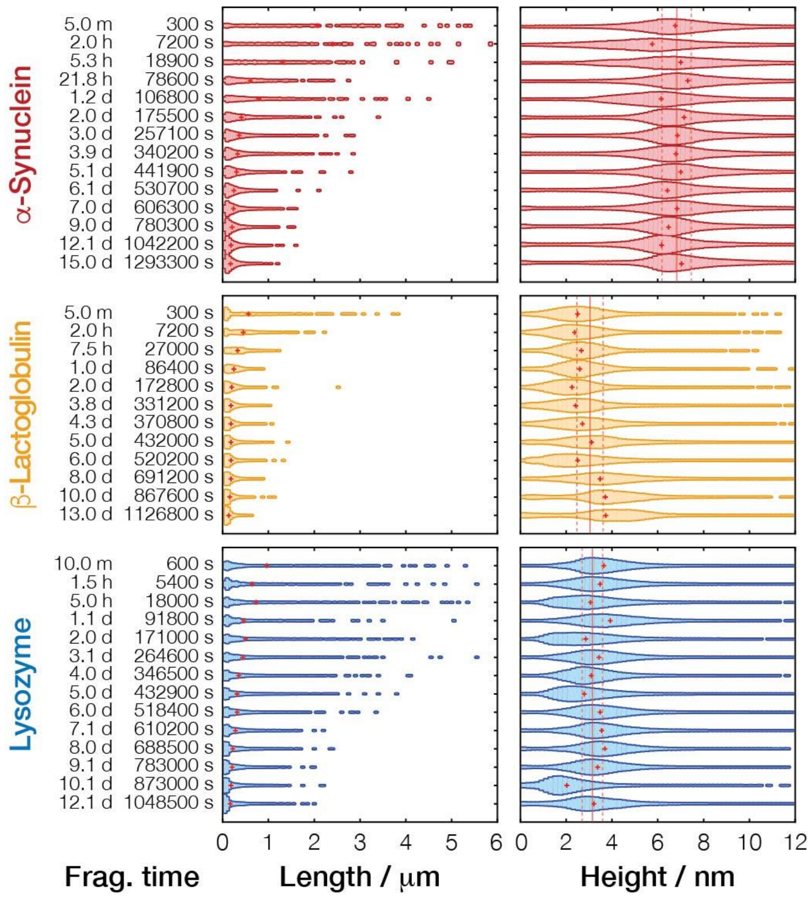

Supporting Figure S2. Fibril length and height distributions extracted from AFM images for fibrils undergoing fragmentation by mechanical stirring. Related to Figure 3. Typical experimental time course with normalized length (left plot of each sample) and height (right plot of each sample) distributions of fibril particles shown as violin plots. The width of the horizontal bars corresponds to the normalised frequencies observed at the length or height indicated by the x-axes. The bars for all samples are shown using the same length and height frequency scales, respectively, to facilitate comparison. The red crosses indicate mean values at each time point and the solid and dashed red lines for height plots indicate mean and standard deviation of all time points taken together, respectively.

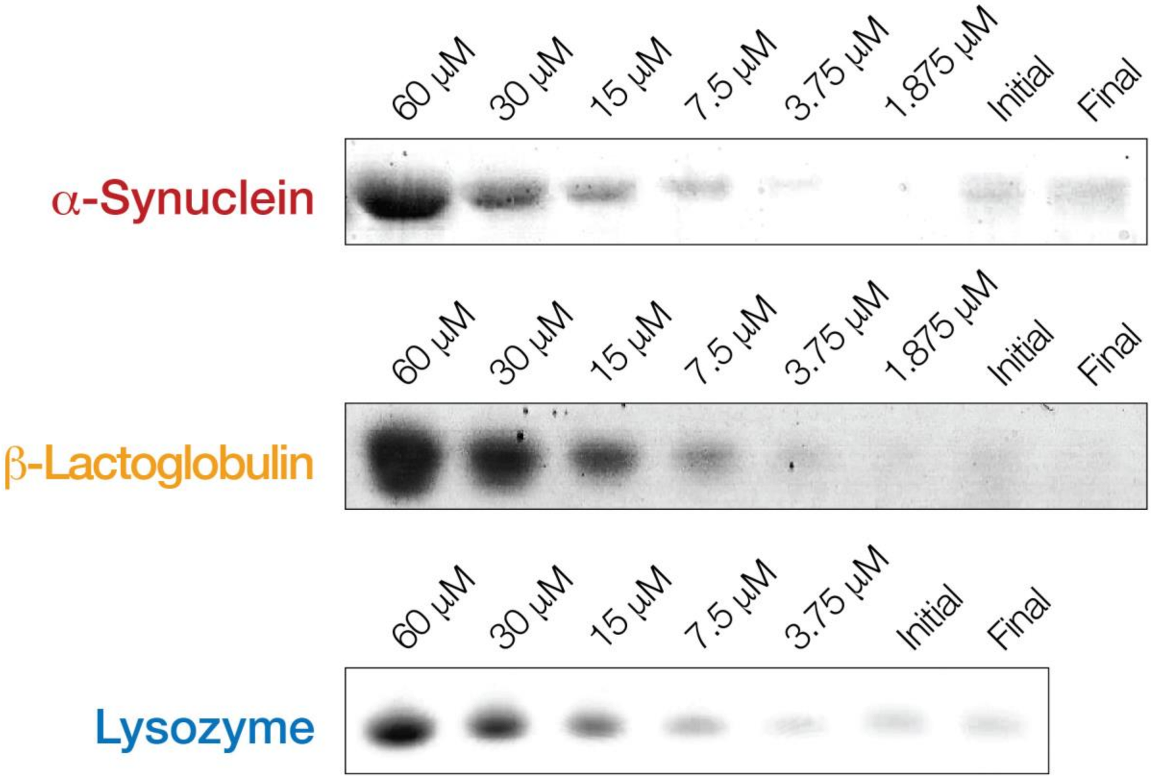

Supporting Figure S3: Residual monomer assay before and after fibril fragmentation time courses. Related to Figure 3. For each fibril type, protein content in the non-pellatable fractions of the initial sample before and Final sample after extended mechanical perturbation were visualised on SDS-PAGE gels together with loading standards of known protein concentrations. The difference in residual monomer concentration (difference between bands in the Initial and Final lanes) were less than 5 % in all cases.

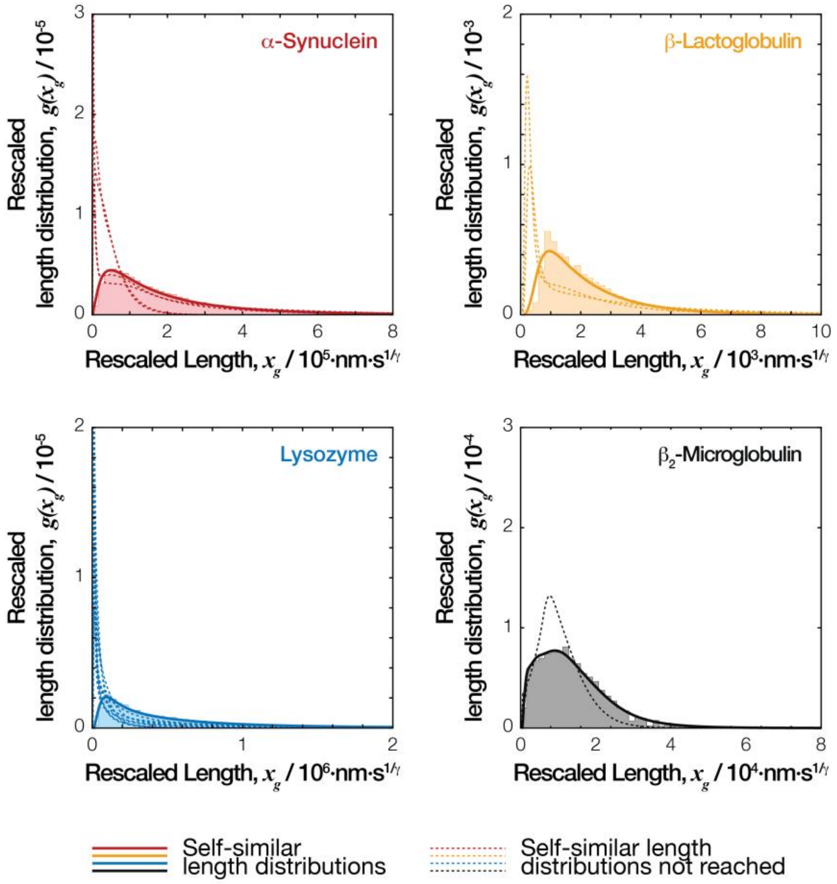

Supporting Figure S4: The self-similar length distribution shape can be obtained from rescaling and averaging of the experimental normalised length distributions. Related to Figure 5. The resealed length distributions $g(x_g)$ calculated with Eq. (5) are shown for each fibril type. For each fibril type analysed, the histograms and bold solid lines are the average of length distributions obtained from AFM imaging analysis that have reached the self-similar length distribution shapes, i.e. distributions at the time points consistent with Eq. (2) in the portion of the experiments represented by the solid lines in **Fig. 5**. for each fibril type. The dashed lines represent distributions from early experimental time-points where self-similarity has not been reached, demonstrating the large deviations from the self-similar distribution shape represented by the bold lines. The lines represent distributions calculated using the kernel density method to reduce clutter and facilitate visualisation and comparison.

SUPPLEMENTARY TABLE

Supporting Table S1: Sample, AFM imaging, and quantitative image analysis statistics. Related to Figure 2 and 3. Image analysis statistics for each sample and time point is shown. List of all raw fibril lengths is available upon request.

Samples		***AFM Imaging***			***Quantitative image Analysis***			
Protein	*Fragmentation Time /s*	*Number of Images*	*Image size / pixels*†	*Scan size / μm*†	*Mean Particle Length / nm*	*Number of Fibril Particles*◊	*Mean Particle Height / nm*	*Number of Pixels* ○
α-Syn	300	6	2048	20	2075.8	653	6.8	49079
	7200	5	2048	20	2402.9	424	5.8	32066
	18900	3	2048	20	1311.6	679	7.0	43259
	78600	2	2048	20	598.9	1350	7.3	53512
	106800	2	2048	20	786.8	1512	6.1	63474
	175500	2	2048	20	406.2	3086	7.1	92632
	257100	2	2048	20	360.1	4087	6.8	110048
	340200	2	2048	20	330.6	4257	6.8	110024
	441900	2	2048	20	301.8	2723	7.0	67278
	530700	2	2048	20	244.7	5064	6.4	110431
	606300	2	2048	20	241.4	6104	6.8	129898
	780300	2	2048	20	208.9	6707	6.5	129581
	1042200	2	2048	20	185.6	6412	6.1	113509
	1293300	2	2048	20	173.6	5346	7.0	90832
	300	5	2048	20	2183.7	507	7.5	37725
	5400	5	2048	20	1756.2	394	7.0	24323
	18000	2	2048	20	1303.7	635	7.8	36938
	91800	2	2048	20	620.7	2293	7.2	87165
	107400	2	2048	20	568.5	2304	7.4	80443

	195900	2	2048	20	439.7	2416	7.2	77497
	259200	2	2048	20	390.5	3264	7.1	93323
	430500	2	2048	20	276.7	4807	6.9	114822
	610200	2	2048	20	224.3	6253	7.1	128690
	691200	2	2048	20	205.2	7541	6.8	145181
	766200	2	2048	20	194.0	5705	6.4	106589
	863700	2	2048	20	181.7	8086	6.5	143206
	1119600	2	2048	20	172.0	9532	6.4	161606
	1219800	2	2048	20	163.6	9470	6.3	154357
	1380900	2	2048	20	158.3	8811	6.8	139488
β-Lac	300	2	2048	20	560.2	496	2.5	28968
	7200	2	2048	20	451.8	854	2.4	40389
	27000	1	2048	20	329.7	751	2.7	26125
	86400	1	2048	20	244.7	1023	2.6	26678
	172800	1	2048	20	200.8	1482	2.3	32010
	331200	2	2048	20	183.0	2637	2.4	52129
	370800	1	2048	20	186.0	1112	2.7	22330
	432000	2	2048	20	185.2	2180	3.1	43588
	520200	2	2048	20	181.1	2190	2.5	42867
	691200	2	2048	20	181.0	1945	3.5	38036
	867600	1	2048	20	163.2	922	3.7	16349
	1126800	1	2048	20	134.2	1138	3.7	16819
	1800	3	2048	20	680.8	898	2.0	63514
	3600	2	2048	20	495.8	977	2.9	50594
	87588	1	2048	20	304.6	2649	3.1	85313
	107712	2	2048	20	310.3	2761	2.9	90538
	182376	3	2048	20	254.9	4664	2.9	126499
	437688	3	2048	20	236.1	3379	3.2	85122
	519876	2	2048	20	233.7	1957	3.6	48815
	624276	4	2048	20	229.9	3511	3.4	86233
	693000	2	2048	20	234.0	1752	3.7	43764
	777312	2	2048	20	237.0	1809	3.7	45739
	1058400	2	2048	20	230.0	830	3.7	20388
	1218960	2	2048	20	213.2	1465	3.2	33468

	1296000	3	2048	20	221.0	2109	3.5	49869
Lyz	300	3	2048	20	1436.6	437	3.1	38771
	1800	2	2048	20	364.7	632	3.4	18882
	3600	2	2048	20	881.6	750	3.1	35831
	7200	2	2048	20	1273.2	610	3.0	41984
	14400	2	2048	20	1103.4	519	3.1	35986
	28800	2	2048	20	1014.9	713	3.0	45954
	86400	2	2048	20	612.0	1500	3.5	79606
	172800	2	2048	20	333.6	2007	2.8	65634
	346600	2	2048	20	242.5	2592	2.9	64844
	432000	2	2048	20	211.3	4020	2.7	88812
	604800	1	2048	20	172.9	4270	2.8	78168
	1123200	2	2048	20	92.5	3419	2.6	35582
	600	3	2048	20	959.5	1402	3.6	69144
	5400	2	2048	20	591.9	1019	3.4	42686
	18000	2	2048	20	720.0	1125	3.0	55008
	91800	1	2048	20	448.8	541	3.9	20366
	171000	2	2048	20	495.9	1934	2.8	79284
	264600	3	2048	20	439.9	3423	3.4	133898
	346500	3	2048	20	351.2	4049	3.1	133400
	432900	3	2048	20	315.0	4798	2.8	145233
	518400	2	2048	20	313.9	3263	3.5	100009
	610200	1	2048	20	275.7	1452	3.5	39803
	688500	1	2048	20	223.1	2100	3.7	47373
	783000	1	2048	20	207.7	2082	3.3	44558
	873000	1	2048	20	181.7	3059	2.0	57672
	1048500	1	2048	20	164.3	2865	3.2	49244
β_2m*	540	16	1024	10	1002.0	468	5.9	36896
	3300	8	1024	10	746.8	515	5.4	29583
	8280	6	1024	10	616.2	650	5.5	29184
	16920	4	1024	10	506.6	603	5.7	22650
	39240	4	1024	10	380.2	859	4.6	25747
	84240	4	1024	10	301.7	1037	5.9	26177
	108000	4	1024	10	266.0	1298	4.8	28612

* Reanalysis of data from Xue and Radford, 2013

† Indicating scan size in μm x μm and image size in pixels x pixels as image aspect ratio was 1 throughout.

◊ Total number of fibril particles quantified for constructing the fibril length distributions.

° Total number of pixel height values in the fibril height distributions for fibril width evaluations.

THEORY

The self-similar division equation

We first explain here the origins of Eq. (1) in the Main Text as well as the assumptions associated with this equation. Let us denote the fibril length distribution *u(t, x)* as the particle concentration of fibrils of length x > 0 at time t, *B(x) ≥ 0* as the division rate constant for fibrils of length *x* (assumed to be independent of time), and *κ(y, dx)* the probability that a dividing fibril of length *y* gives rise to two fibrils of size *x* and *y* − *x* (Fig. 1b). The *κ*, often called fragmentation kernel, is nonnegative and satisfies the following properties:

$$\int_0^y \kappa(y, dx) = 1, \qquad \kappa(y, x > y) = 0, \qquad \kappa(y, x) = \kappa(y, y - x)$$

Eq. (S1)

The last property above is a symmetry property linked to the assumption that we consider only division into two daughter fibrils for each microscopic step, and the fibrils are isotropic along the axis of the filament so the division rate only depends on the length of the resulting two fibrils (**Fig. 1c** and **1d**). The first two properties of Eq. (S1) express that *κ(y, dx)* is a normalised probability density function, and that daughter fibrils post-division are always shorter than their mother fibril. The time dependent concentration of fibrils *u(t, x)* then satisfies the following equation:

$$\frac{\partial}{\partial t}u(t,x) = -B(x)u(t,x) + 2\int_{y=x}^{\infty} \kappa(y,x)B(y)u(t,y)dy, \qquad u(0,x) = u_0(x)$$

Eq. (S2)

where $u_0(x)$ is the initial length distribution of fibrils. Equation (S2) is the continuous division equation, which describes the evolution $\frac{\partial}{\partial t}u(t,x)$ of the fibril particle concentrations in the fibril length

distribution $u(t, x)$ with respect to time t. It states that fibrils of a given length x in the sample distribution will be consumed with a rate $B(x)$ when they divide into smaller daughter fibrils, and that fibrils of the same length x may also appear in the sample distribution each time a fibril of size $y > x$ divides into two fibrils of size x and $y - x$. Let us denote the total initial mass of fibrils as $\rho = \int_0^\infty x u_0(x)dx$. Since the mass is conserved through time: $\int_0^\infty x u(t,x)dx = \rho$. We also assume, in line with previous theoretical (Hill, 1983) and experimental results (Xue and Radford, 2013), that the division rate constant is given by a power law:

$$B(x) = \alpha_0(\alpha x)^\gamma, \qquad \alpha > 0, \qquad \gamma > 0$$

Eq. (S3)

and that the site where a fragmenting fibril of size y breaks down only depend on the relative

position of its site along the mother fibril, defined by the ratio x/y where x is the length of one of the two daughter fibrils. This property is called a "self-similar" division and is translated mathematically with fragmentation kernel κ as the following:

$$\kappa(y,x) := \frac{1}{y}\kappa_0\left(\frac{x}{y}\right)$$

Eq. (S4)

where the properties described by Eq. (S1), when transferred to the probability density κ_0, and with $z = \left(\frac{x}{y}\right)$, satisfies the following:

$$\int_0^1 \kappa_0(z)dz = 1, \qquad \kappa_0(z > 1) = 0, \qquad \kappa_0(z) = \kappa_0(1 - z)$$

Eq. (S5)

Two important examples may be viewed as special cases of self-similar fragmentation kernels above. The first one is the case of division of uniform probability: the parent fibril can break at any site along its length with an equal probability, so that $\kappa_0\left(\frac{x}{y} \in (0,1)\right) = 1$. The second special division case is

sometimes referred to as the "equal mitosis case" from its roots in describing cellular divisions, where the parent fibril divides exactly at the middle, so that we have a Dirac delta function at $\kappa_0\left(\frac{1}{2}\right)$: $\kappa_0\left(\frac{x}{y}\right) = \delta_{\frac{x}{y}=\frac{1}{2}}$. Using all of the properties and assumptions above, the continuous division equation Eq. (S2) then becomes:

$$\frac{\partial}{\partial t}u(t,x) = -\alpha_0(\alpha x)^{\gamma}u(t,x) + 2\int_{y=x}^{\infty}\frac{1}{y}\kappa_0\left(\frac{x}{y}\right)\alpha_0(\alpha x)^{\gamma}u(t,y)dy$$

Eq. (S6)

which is equation Eq. (1) in the Main text.

Long-time behaviour of the continuous division equation

For our continuous division equation Eq. (1) and (S6), it has been proven in (Escobedo et al., 2005) that for long times, there exists a unique probability density function *g* and a constant $C_g > 0$ such that:

$$u(t,x) \underset{t\to\infty}{\longrightarrow} C_g t^{\frac{2}{\gamma}} g(x_g), \qquad x_g = x t^{\frac{1}{\gamma}}$$

Eq. (S7)

The constant C_g is introduced to ensure mass conservation, which holds for any time *t*. Eq. (S7) means that for large times, the probability density *u* tends towards a specific distribution shape *g* after variable rescaling. Moreover, the function *g* is defined as the unique solution to the following equation:

$$x_g\frac{dg(x_g)}{dx_g} + (2 + \alpha\gamma {x_g}^{\gamma})g(x_g) = 2\alpha\gamma\int_{y_g=x_g}^{\infty}\frac{1}{y_g}\kappa_0\left(\frac{x_g}{y_g}\right)y_g^{\gamma}g(y_g)dy_g\,, \qquad \int_0^{\infty}g(y_g)dy_g = 1$$

Eq. (S8)

We can then compute the constant C_g as the following:

$$\int_0^{\infty} xu(t,x)dx = \rho = C_g \int_0^{\infty} t^{\frac{2}{\gamma}} xg\left(xt^{\frac{1}{\gamma}}\right)dx = C_g \int_0^{\infty} x_g g(x_g)dx_g \quad \Rightarrow \quad C_g = \frac{\rho}{\int_0^{\infty} x_g g(x_g)dx_g}$$

Eq. (S9)

We then relate these results to our experimental measurements. First, since we measure at successive time points small aliquots taken from the fibril samples, these samplings may be viewed as measurements of the length distribution of the fibril sample at time points *t*. We also do not measure directly *u(t, x)*, since the total number of fibrils is not known *a priori* for each time point. Instead, we measure the normalised length distribution *f(t, x)* as described below. Using Eq. (S7-S9), we then have the following:

$$\int_0^{\infty} u(t,x)dx \xrightarrow[t\to\infty]{} C_g \int_0^{\infty} t^{\frac{2}{\gamma}} g\left(xt^{\frac{1}{\gamma}}\right)dx = C_g t^{\frac{1}{\gamma}} \int_0^{\infty} g(x_g)dx_g = C_g t^{\frac{1}{\gamma}}$$

Eq. (S10)

We can define $f(t,x)$ as the normalised fibril length distribution that can be assessed using the experimental image data:

$$f(t,x) = \frac{u(t,x)}{\int_0^{\infty} u(t,x)dx}$$

Eq. (S11)

Using this definition of *f(t, x)* from, we then have:

$$f(t,x) \xrightarrow[t\to\infty]{} \frac{C_g t^{\frac{2}{\gamma}} g(x_g)}{C_g t^{\frac{1}{\gamma}}} = t^{\frac{1}{\gamma}} g(x_g), \qquad x_g = xt^{\frac{1}{\gamma}}$$

Eq. (S12)

which is equation Eq. (3) of the main text. Next, defining the average length of fibrils *μ(t)* as the experimentally tractable time-dependent mean length of the fibril length distribution defined as:

$$\mu(t) \;=\; \int_0^\infty x \cdot f(t,x)dx$$

Eq. (S13)

We have the following relationship:

$$\mu(t) := \int_0^\infty xf(t,x)dx \underset{t\to\infty}{\longrightarrow} \int_0^\infty xt^{\frac{1}{\gamma}} g\left(xt^{\frac{1}{\gamma}}\right)dx = t^{-\frac{1}{\gamma}} \int_0^\infty x_g g(x_g)dx_g = Ct^{-\frac{1}{\gamma}},$$

$$C = \int_0^\infty x_g g(x_g)dx_g$$

Eq. (S14)

which is the relationship between the average length of fibrils and time *t* in equation Eq. (2) of the main text.

Estimating the division parameters α and γ

We first estimate *γ* by fitting a modified version of Eq. (2) to the average lengths *μ(t)* estimated from the experimentally observed fibril length distributions for sufficiently long times (see Eq. S22 in Transparent Methods). Then, we estimate *α* from *γ* and *g* using Eq. (S8). Integration of Eq. (S8) yields:

$$\int_0^\infty x_g \frac{dg(x_g)}{dx_g}dx_g + \int_0^\infty 2g(x_g)dx_g + \alpha\gamma \int_0^\infty {x_g}^\gamma g(x_g)dx_g = 2\alpha\gamma \int_0^\infty \int_{y_g=x_g}^\infty \frac{1}{y_g}\kappa_0\left(\frac{x_g}{y_g}\right) y_g^\gamma g(y_g)dy_g\, dx_g$$

Eq. (S15)

We can integrate Eq. (S15) by parts the first term, and we use Fubini's theorem to invert the integral order in the last term:

$$-\int_0^\infty g(x_g)dx_g + \int_0^\infty 2g(x_g)dx_g + \alpha\gamma \int_0^\infty {x_g}^\gamma g(x_g)dx_g = 2\alpha\gamma \int_0^\infty \int_{x_g=0}^{y_g} \frac{1}{y_g}\kappa_0\left(\frac{x_g}{y_g}\right) y_g^\gamma g(y_g)dx_g\,dy_g$$

Eq. (S16)

We then use the fact that g is normalised, $\int_0^\infty g(y_g)dy_g = 1$, and change the variable x_g to $z = \left(\frac{x_g}{y_g}\right)$ to obtain:

$$1 + \alpha\gamma \int_0^\infty {x_g}^\gamma g(x_g)dx_g = 2\alpha\gamma \int_0^\infty \int_{z=0}^{1} \kappa_0(z) y_g^\gamma g(y_g)dz\,dy_g$$

Eq. (S17)

Using the property $\int_0^1 \kappa_0(z)dz = 1$ from Eq. (S1), we obtain:

$$1 = \alpha\gamma \int_0^\infty {x_g}^\gamma g(x_g)dx_g$$

Eq. (S18)

To relate α directly to the experimentally characterised *f(t,x)* rather than on g, we multiply the equation Eq. (S12), i.e. Eq. (3) of the main text, by x^γ and integrate it to obtain the following:

$$\int_0^\infty x^\gamma f(t,x)dx \underset{t\to\infty}{\longrightarrow} \int_0^\infty x^\gamma t^{\frac{1}{\gamma}} g\left(x t^{\frac{1}{\gamma}}\right)dx = \int_0^\infty x_g^\gamma t^{-1} g(x_g)dx_g$$

Eq. (S19)

Rearranging Eq. (S18) and using Eq. (S19), we obtain:

$$\alpha = \frac{1}{\gamma}\frac{1}{\int_0^\infty {x_g}^\gamma g(x_g)dx_g} \underset{t\to\infty}{\longrightarrow} \frac{1}{\gamma}\frac{t^{-1}}{\int_0^\infty x^\gamma f(t,x)dx}$$

Eq. (S20)

Therefore, we get the following relationship:

$$\alpha \approx \frac{1}{\gamma} \frac{t^{-1}}{\int_0^\infty x^\gamma f(t,x)dx}, \qquad t \gg t_0$$

Eq. (S21)

which is used to estimate α from experimental data. For more details, we also refer the interested reader to Doumic et al., 2018, and more specifically to Lemma 1 and Eq. (3.3) in this reference.

TRANSPARENT METHODS

Preparation of protein monomers

Hen egg white Lyz and bovine β-Lac proteins were both purchased from Sigma-Aldrich and used with no further purification. Production and purification of human α-Syn monomers was achieved according to the method of Cappai et al (Cappai et al., 2005), with the addition of a stepped ammonium sulphate precipitation (30% to 50%) step prior to anion exchange chromatography. The protein was buffer exchanged using PD10 desalting column (GE Healthcare) prior to loading onto the anion exchange resin.

In vitro formation of amyloid fibril samples

The conversion of Lyz and β-Lac to amyloid fibres was achieved under acidic and heated conditions. Both proteins were dissolved in 10 mM HCl to a concentration of 15mg/ml and then incubated for 4 hr at 25 °C. The resulting solutions were filtered through a 0.2 μm syringe filter and diluted to a concentration of 10mg/ml (Lyz = 699 μM and β-Lac = 547 μM). 500 μl aliquots were then heated without agitation for differing periods of time, with Lyz heated at 60 °C for 2 days and β-Lac heated at 90 °C for 5 hr. α-Syn fibrils were formed by buffer exchange of purified monomers into fibril forming buffer (20mM Sodium phosphate, pH7.5) using a PD-10 column (GE Healthcare). The resulting α-Syn solution was passed through a 0.2 μm syringe filter. Protein concentration was subsequently determined via absorbance at 280nm, and the sample solution were diluted to 300 μM and incubated at 37 °C in a shaking incubator with agitation set at 200 rpm for at least two weeks.

Controlled fibril fragmentation through mechanical perturbation

Parent fibril solutions were diluted to 120 µM using the appropriate fibril forming buffer for each protein in a snap cap vial containing an 8 x 3 mm PTFE coated magnetic stirrer bar and then subjected to stirring at 1000 rpm on an IKA squid stirrer plate with digital speed display. At appropriate time points, small aliquots of the fibril samples were removed, diluted with fibril forming buffer (deposition concentration for α-synuclein is 0.48 µM, β-lactoglobulin is 0.6 µM and Lyz is 6 µM), and 20 µl were immediately taken and incubated for 5 min on freshly cleaved mica surfaces (Agar Scientific F7013). The mica surfaces were subsequently washed with 1 ml of syringe filtered (0.2 µm) mQ H_2O and dried under a gentle stream of N_2(g).

Determination of residual monomer concentration

Residual monomer concentration for each fragmentation sample were measured using SDS-PAGE after centrifugation (75000 rpm, 15 min) with 100 µl of the 120 µM fragmentation reaction and 100 µl of 120 µM non-fragmented parent fibrils samples. The top 10µl of the solutions were then removed and treated with 4x loading dye and boiled at 95 °C for 5 min (Lyz samples were heated to 65 °C and beta-mercaptoethanol was not added due to decomposition of samples). The samples were then run against a serial dilution of monomeric protein standards on either a Tris-Tricine gel or a 15% Tris-Glycine gel at 180V and subsequently stained with Coomassie blue. Analysis of the protein bands was carried out by densitometry for comparison of bands to the serial dilution bands.

AFM imaging and image analysis

The fibril samples were imaged on a Bruker Multimode 8 scanning probe microscope with a Nanoscope V controller, using the ScanAsyst peak-force tapping imaging mode. Bruker ScanAsyst probes (Silicone nitride tip with tip height = 2.5-8 µm, nominal tip radius = 2 nm, nominal spring constant 0.4 N/m and nominal resonant frequency 70 kHz) were used throughout. Multiple 20 µm x 20 µm areas of the surface were scanned at a resolution of 2048 x 2048 pixels. The images were then processed and flattened using Bruker Nanoscope Analysis software to remove tilt and bow. The images were then

imported into Matlab, where length of individual fibril particles was measured. The sample length and height distributions were obtained as previously described (Xue, 2013; Xue et al., 2009). For the fibril length distributions, any length-dependent bias in a deposition for imaging or during the fibril tracing step of image analysis was taken into account as previously described (Xue et al., 2009).

Data analysis of fibril division properties

The normalised length distribution of the fibril samples measured by AFM at time t, $f(t, x)$, is linked to the concentration of fibrils solution $u(t, x)$ in Eq. (1) by the relation in Eq. (SI.11). Mean lengths for each time point $\mu(t)$ were calculated from the experimental $f(t, x)$ distributions and Eq. (2) and (4) where used to first extract γ from the datasets. Because some unknown number of experimentally measured length distributions at early time points in the experiments may not have sufficiently reached the self-similar distribution at the asymptotic line (i.e. where Eq. (2) does not apply), we fit the following equation Eq. (4) to the average length as function of time data instead of Eq. (2) directly in order to estimate the number of experimental time points consistent with the self-similar distribution shape objectively without human input:

$$\begin{cases} \mu(t) = C \cdot t^{-1/\gamma}; & t > t_s \\ \mu(t) = C \cdot t_s^{-1/\gamma}; & t \leq t_s \end{cases}$$

Eq. (S22)

Eq. (4) was fit to the average length $\mu(t)$ as function of time t, with C and t_s as parameters individual to each experimental dataset and γ as a global parameter for datasets from the same fibril type. Subsequently, the $g(x_g)$ and α values were calculated with Eq. (3) and (SI.21), respectively, both using γ calculated above and experimental normalised length distributions $f(t, x)$ where $t > t_s$. For both the $g(x_g)$ distributions and α values, averages were obtained for each fibril type. The self-similar distribution shapes $g(x_g)$ were used to calculate length distribution at any time using the reverse of Eq. (3). The α and γ values were used to calculate the division rate constant $B(x) = \alpha_0(\alpha x)^\gamma$ for fibrils of

any length x. Supplementary information section contains further information on the mathematical considerations of our division model.

Direct numerical simulation of fibril division processes

To validate the α and γ values obtained from our analysis, direct numerical simulations to calculate the time evolution of the fibril length distributions were carried out by numerically solving the full ODE system describing the master equation mostly as described in Xue and Radford, 2013 but with a few modifications. Firstly, numerical integrations of the master equation were solved for fibril species containing up to 30,000 instead of 20,000 monomeric units in order to retain concentration errors introduced by numerical inaccuracy and truncation of larger species to <1%. Secondly, the number of division sites was assumed to be equal to the number of monomers-1 and the unit used for the length of fibrils was interconverted in the simulations from nanometres (x in [nm] units) to the number of monomers (i number of monomers) using the numbers of monomers per nm length unit N_l (Xue and Radford, 2013) as conversion factor. Subsequently, assuming that division sites along the fibrils operate independently, the microscopic rate constant on per division site basis is $B(i)\kappa_0$ divided by the number of monomers-1. Thirdly, as $g(x_g)$ shape for Lyz and α-Syn fibril divisions suggest a κ_0 function that result in similar division rates in the fibril centre and fibril edge, simulations for these two fibril types were carried out using Eq. S6 in Xue and Radford 2013 instead of Eq. S8. Finally, the experimental distribution at the first time-points (including all the experimental noise) were directly used as the initial distribution (dashed lines in **Fig. 6**) instead of parameterised distributions (Xue et al., 2009) in the simulations to illustrate the fact that our model has shown that the self-similar distribution shape will be reached independently of the initial length distribution.
